# A Highly Permeable and Three-Dimensional Integrated Electronic System for Wearable Human–Robot Interaction

**DOI:** 10.1007/s40820-025-01974-z

**Published:** 2026-01-03

**Authors:** Wenqiang Wang, Zebang Luo, Xingge Yu, Xiaojia Yin, Li Xiang, Anlian Pan

**Affiliations:** 1 Key Laboratory for Micro-Nano Physics and Technology of Hunan Province, State Key Laboratory of Chemo/Biosensing and Chemometrics, College of Materials Science and Engineering, Hunan Institute of Optoelectronic Integration, Hunan University, Changsha, 410082 People’s Republic of China; 2 School of Physics and Electronics, Hunan Normal University, Changsha, 410081 People’s Republic of China

**Keywords:** Permeable electronics, Stretchable electronics, Multilayer electronic system, Gesture recognition, Vertical interconnect access (VIA)

## Abstract

Permeable electronics promise improved physiological comfort, but remain constrained by limited functional integration and poor mechanical robustness. Here, we report a three-dimensional (3D) permeable electronic system that overcomes these challenges by combining electrospun SEBS nanofiber mats, high-resolution liquid metal conductors patterned via thermal imprinting (50 μm), and a strain isolators (SIL) that protects vertical interconnects (VIAs) from stress concentration. This architecture achieves ultrahigh air permeability (> 5.09 mL cm^−2^ min^−1^), exceptional stretchability (750% fracture strain), and reliable conductivity maintained through more than 32,500 strain cycles. Leveraging these advances, we have integrated multilayer circuits, strain sensors, and a three-axis accelerometer to achieve a fully integrated, stretchable, permeable wireless real-time gesture recognition glove. The system enables accurate sign language interpretation (98%) and seamless robotic hand control, demonstrating its potential for assistive technologies. By uniting comfort, durability, and high-density integration, this work establishes a versatile platform for next-generation wearable electronics and interactive human–robot interfaces.

**Supplementary Information:**

The online version contains supplementary material available at 10.1007/s40820-025-01974-z.

## Highlights

- *Breathable and Stretchable 3D Electronics* Electrospun SEBS nanofiber mats combined with sub-50 μm liquid metal patterning yield ultrahigh permeability (5.09 mL cm^−2^ min^−1^, 2520 g m^−2^ day^−1^) and mechanical robustness (750% strain), ensuring zero skin irritation after 1 week.
- *Stable Vertical Interconnects* Strain isolators (SIL) decouple substrate deformation from vertical interconnects, maintaining conductivity under 750% strain and >32,500 cycles, surpassing conventional multilayer systems (<250% strain).
- *Gesture Recognition Assistive Glove* A wireless glove integrating 5 strain sensors, a three-axis accelerometer, and CNN-based learning (98% accuracy) enables real-time robotic hand control, with direct relevance to rehabilitation, prosthetics, and human–robot collaboration.

**Supplementary Information:**

The online version contains supplementary material available at 10.1007/s40820-025-01974-z.

## Graphical abstract

**Figure:**
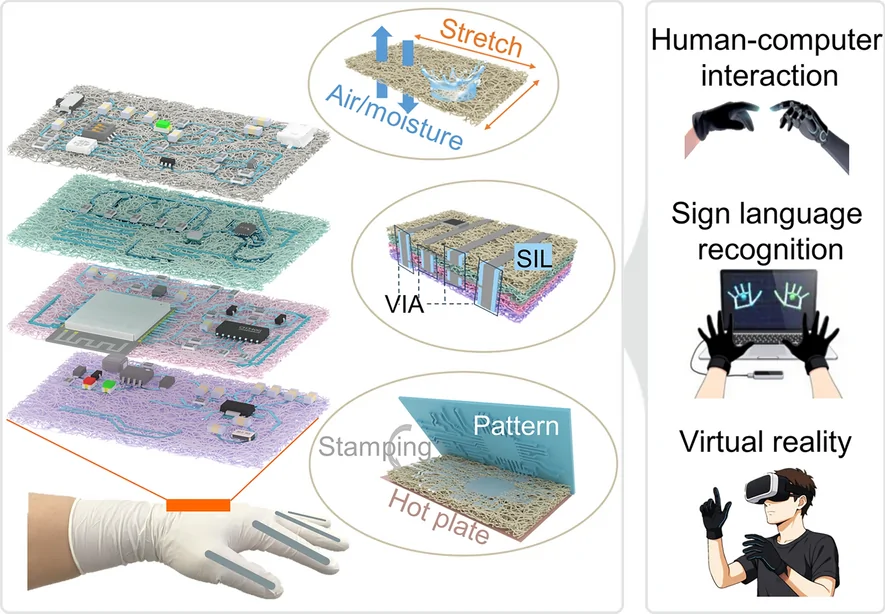

## Introduction

Flexible and stretchable electronics have attracted broad attention across diverse applications, such as wearable electronics [1–4], soft robotics, virtual reality (VR), and human–computer interaction, which could enable seamless interface between the electronic systems and the human body, thereby conforming to complex geometries and enduring mechanical deformations [5, 6]. Despite the significant advances in improving the mechanical and electrical properties of these flexible and stretchable devices [7–9], critical consideration of the physiological conditions of the human body should also be required in the practical clinical implementation [10]. These imperatives have spurred the development of permeable electronics in recent years [11–14], which referred as wearable technologies that maintain electronic functionality while minimizing thermo-physiological disturbance with the human body [15].

The permeable electronics are generally based on porous, nanofibrous, mesh, or textile substrates [16–19]. These structures enhance comfort and reduce irritation during long-term wear [20, 21]. However, compared with conventional wearable electronics that integrate complex circuits for acquisition, processing, and storage [22], permeable devices remain limited to basic components such as electrodes, sensors, and antennas. Their low integration density and functional simplicity hinder practical deployment. Three-dimensional (3D) integration improves device density and complexity compared with single-layer systems [23–25]. Yet, building robust 3D permeable systems faces challenges in materials, fabrication, and mechanics [26]. On one the hand, conductive traces on porous substrates are usually printed inks/pastes [27, 28], with resolution often in the millimeter range, restricting integration density. Photolithography offers higher resolution [29], but the stretchability of patterned metals under large strains remains limited [30]. On the other hand, for 3D integration systems, the vertical interconnect accesses (VIAs) between different layers are often made by laser drilling [24, 31], but precise control is difficult [32], and modulus mismatch at VIA interfaces causes stress concentration and electrical failure under strain [33, 34].

In this article, we address the aforementioned challenges though a synergistic approach involving manufacturing process and mechanical design, which enable a highly integrated, permeable, and stretchable multilayer electronic system to serve as a versatile platform for the integration of various wearable electronic components, particularly for applications in interactive robotics. All functional electronic components within this system are seamlessly integrated onto a highly permeable substrate crafted from electrospun fibers and interconnected via stretchable liquid metal conductors. Distinguished from the reported conductors manufacturing on permeable substrates, which are challenging to achieve both stretchability and high-resolution patterning, a thermal imprinting technique was applied here to achieve high-precision manufacturing of interconnecting conductors on the permeable and stretchable substrates, with a minimum feature size as fine as 50 µm. Meanwhile, we have engineered a strain isolators (SIL) to redistribute mechanical stresses at vertical interconnect accesses (VIAs) to maintain stable electrical connectivity in 3D integrated systems. To demonstrate the capabilities of the system, we designed a stretchable and permeable liquid metal-based strain sensor and integrated it with the 3D system to form a fabric glove. The gloves not only exhibit superior permeability and stretchability but also provide highly sensitive gesture recognition capabilities (with 98% accuracy). These attributes make them suitable for long-term dynamic monitoring of hand movements and have potential applications in controlling robotic hands and aiding in sign language translation.

## Experimental Section

### Materials

SEBS (KRATON G1652) was purchased from Changhong Plastic Co., Ltd. (Dongguan, China). Tetrahydrofuran (THF, AR, 99.0%) was obtained from Aladdin Bio-Chem Technology Co., Ltd. (Shanghai, China). *N*,*N*-Dimethylformamide (DMF, ACS, 99.8%) was obtained from Macklin Biochemical Technology Co., Ltd. (Shanghai, China). EGaIn (melting point: 11 °C, conductivity: 3.46 × 10^6^ S m^−1^, surface tension: 0.718 N m^−1^) was procured from Huatai Metal Materials Technology Co., Ltd. (Dongguan, China). PDMS (SYLGARD 184, Dow Corning) was prepared by mixing pre-polymer and cross-linker at a mass ratio of 10:1. SEBS films was prepared by dissolving the SEBS polymer with a weight ratio of 10 wt% in the mixed solvent (tetrahydrofuran/dimethylformamide = 1:1). Liquid PDMS and SEBS were degassed in a vacuum desiccator to remove bubbles.

### Preparation of SEBS Fiber Mat

The SEBS polymer was dissolved at a concentration of 10 wt% in a mixed solvent of tetrahydrofuran/dimethylformamide (1:1 by volume), followed by magnetic stirring at 80 °C for 5 h to obtain a transparent and viscous spinning solution. The solution was then transferred to a 5 mL syringe for electrospinning (Fig. S7a**)**. The electrospinning conditions were as follows: applied voltage of 11.3 kV, solution feed rate of 2 mL h^−1^, and a fiber collection distance of 25 cm. The process was carried out at room temperature. The obtained SEBS fiber mat was dried at 80 °C for 2 h to remove residual solvents and facilitate peeling from the tin foil substrate.

### Fabrication of Multilayer Stacked Stretchable and Breathable Liquid Metal Circuits

First, a release agent is sprayed onto the surface of a 4-inch wafer and heated to form a cured release layer. Next, a static electrospinning process is used to fabricate a porous SEBS fiber mat with a thickness of 0.5 mm from an SEBS solution. Subsequently, a template with pre-designed circuit layout is fabricated using 3D printing technology and placed on the surface of the SEBS fiber mat. The composite structure is then subjected to a patterning pre-treatment in a thermal imprinting apparatus at 125 °C and 9 kPa, which induces selective wetting areas on the SEBS fiber mat surface to facilitate the subsequent patterned deposition of liquid metal.

After the substrate pre-treatment, electronic components are precisely positioned at the pre-defined locations, and their pins are fixed using low-temperature conductive solder. Liquid metal is then coated onto the surface of the thermal imprinting substrate to form interconnected circuit pathways. To further ensure interfacial reliability, an adhesive layer was applied to immobilize the components on the porous nanofiber substrate (Fig. S1). To further enable vertical interconnection, small through-holes are created at specific locations on the circuit using a mini punching machine. SIL are fixed at the through-hole positions, and liquid metal is filled to ensure interlayer electrical connections. The same process is repeated for the fabrication of additional multilayer circuits.

Upon completion of all layers, the stack is detached from the wafer surface and stacked in sequence, ensuring precise alignment of interlayer through-holes. To enhance the interlayer adhesion, an atomized tetrahydrofuran (THF) solution is used to treat the stacking interfaces, causing the top layer fibers to swell and achieving a tight bond. The complete circuit schematic is shown in Fig. S19. Finally, the entire circuit is encapsulated using a breathable, waterproof SEBS fiber mat to ensure stable operation under complex conditions.

### Fabrication of the Strain Isolators (SIL)

Figure S24 presents the schematic diagram of the production process of strain isolators (SIL). First, a negative mold is fabricated based on the 3D model of the SIL (Fig. S17a). Before use, a release agent is applied to the mold and heated at 80 °C to form a thin film. Subsequently, a 20 wt% SEBS solution is injected into the mold and placed in a vacuum oven for degassing and curing. Finally, through-holes are created in the SIL using a small punching machine to enable interlayer electrical connections in the circuit.

### Finite Element Analysis

Finite element analysis (FEA) of the mechanical properties of the core–shell substrate was performed using Ansys Workbench 2023 R1 with CAM analysis. SEBS was modeled as a hyperelastic material using the Mooney–Rivlin constitutive model with material coefficients C10 = 2.93 × 10^5^, C01 = 1.77 × 10^5^, and D1 = 0. The model dimensions were consistent with those of the actual device to ensure the accuracy of the simulation results.

### Characterization

Images of the surface morphology of the samples were collected by SEM (IGMAHD, Carl Zeiss). The mechanical properties of the mat were tested using a tensile tester (FlexTest-L-L, Hunan NanoUp Electronics Technology Co., Ltd.). The electrical properties of the mat were investigated by a digital source measure unit (S300B, Wuhan PRECISE Instrument Co., Ltd.). The thermal images of the 8-layer stretchable heater were captured using the InfiRay XP09 V2. Both air permeability and moisture permeability tests were performed at constant temperature (20 °C) and humidity (53%). The air permeability test was conducted using a custom-built apparatus based on the pressure differential method (Fig. S25). The membrane sample was securely fixed in a sealed fixture equipped with inlet and outlet ports. The fixture's inlet was connected to a nitrogen gas cylinder via a pressure regulation module, while the outlet was coupled to a water-filled bottle and a graduated cylinder. Nitrogen gas, regulated by a pressure-reducing valve, entered the fixture through the inlet at a controlled pressure of 1 kPa, permeated through the membrane, and exited via the outlet into the water-filled bottle and measuring cylinder. The volume of water displaced in the graduated cylinder was measured over a predetermined time interval to calculate the membrane's air permeability. Moisture permeability tests were performed according to the E96/E96M-13 standard with the cup method. The testing duration was 48 h. The contact angle of the SEBS fiber mat was measured by a contact angle measuring instrument (SDC-200S, Siwaka Precision Instrument Co., Ltd.).

## Results and Discussion

### Design Concepts for 3D Permeable and Stretchable Electronic Platform

**Fig. 1:**
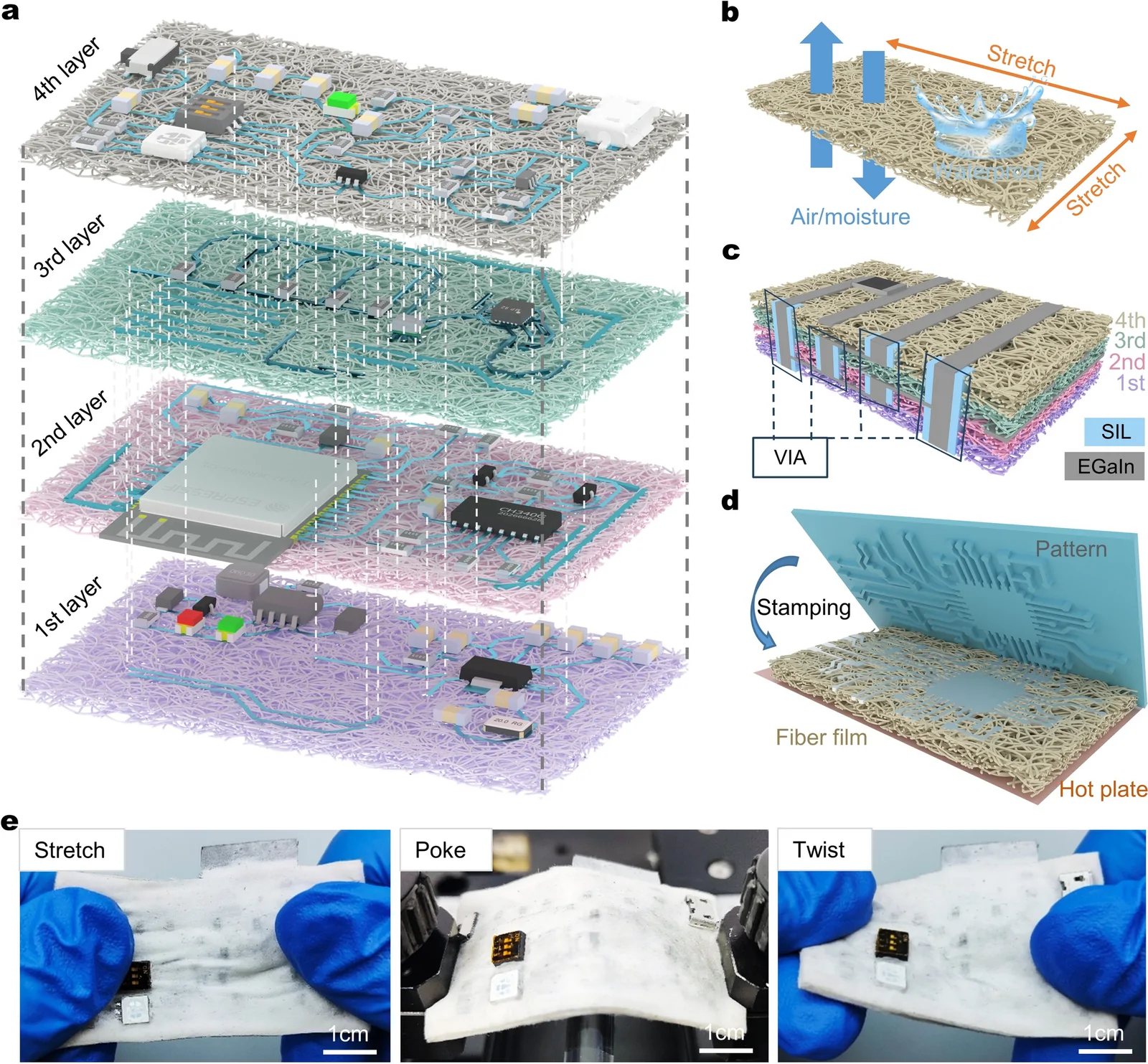
Architecture of the 3D permeable electronic platforms. **a** Exploded schematic of the 3D permeable electronic platform, composed of four vertically stacked layers. Dashed lines indicate vertical interconnect accesses (VIAs) that establish interlayer electrical connections. **b** Illustration of stretchable, permeable, and waterproof properties in the electrospun SEBS fiber mat. **c** Schematic of VIAs integrated with strain isolators (SILs) in multilayer configurations. **d** Patterned liquid metal in-plane interconnects fabricated via thermal stamping techniques. **e** 3D permeable system maintains mechanical compliance and durability under mechanical stress, including 30% tensile strain, localized punctures, and 30-degree torsional deformation

Figure 1a illustrates the fundamental structure and circuit layout of the proposed 3D electronic platform, which comprises four vertically stacked layers. Each layer incorporates both active (e.g., amplifiers, RF devices) and passive (e.g., resistors, capacitors, inductors, and sensors) components, interconnected via patterned liquid metal traces to ensure stretchability. The electronic components were fixed at the pre-defined positions and connected using low-temperature conductive solder. To further ensure interfacial reliability, a glue was applied to immobilize the components on the porous nanofiber substrate (Fig. S1). Electrical stability tests conducted on representative 10 Ω and 100 Ω resistors demonstrated negligible resistance variation after 9000 cycles at 100% tensile strain (Fig. S2), confirming that the component–substrate bonding maintains excellent reliability under repeated bending and stretching. The vertical integration is achieved through a layer-by-layer assembly process, culminating in a highly compact and multifunctional 3D electronic system, accommodating to 140 sub-components. Key design considerations of this platform revolve around three core aspects: (i) Permeability: To address physiological comfort during prolonged wear, each layer of the 3D integrated system is supported by an electrospun nanoporous SEBS fiber mat (Fig. 1b). This substrate not only provides exceptional mechanical flexibility but also ensures effective gas and moisture permeability (Movie S1), mitigating potential skin irritation and thermal discomfort. By leveraging a porous nanofiber mat, the platform maintains a highly permeable interface, significantly outperforming conventional flexible substrates such as polyimide (PI) and polydimethylsiloxane (PDMS). Benefiting from the inherent hydrophobicity of SEBS material and its nanoporous structure (with pore sizes around 19.34 μm, as shown in Fig. S7e), the film exhibits excellent waterproof performance. Water resistance tests demonstrate that water droplets (typically larger than 19.34 μm in diameter) form distinct spherical beads on the film surface (Fig. S3), establishing a stable solid–liquid–gas hydrophobic interface. Notably, when water molecules exist in vapor form (with a kinetic diameter significantly smaller than 19.34 μm), they can penetrate the nanoscale pores, effectively balancing the requirements of waterproofness and breathability. (ii) Mechanical robustness of vertical interconnects: A persistent challenge in multilayer stretchable systems is maintaining reliable vertical electrical interconnections (VIAs) under dynamic deformations. To mitigate VIA failure caused by mechanical strain, we introduce a strain isolators (SIL), as depicted in Fig. 1c. The SIL effectively shields VIAs from excessive strain, ensuring stable electrical performance under repeated stretching and bending. This strategy addresses a longstanding limitation in stretchable 3D electronics, where VIA integrity is often compromised due to substrate deformation and interfacial stress mismatches. (iii) High-resolution in-plane electrical interconnects: To achieve high-density integration of electronic components within each layer, we employ eutectic gallium–indium (EGaIn) liquid metal as the stretchable conductor. EGaIn exhibits excellent electrical conductivity and mechanical deformability; however, its high surface tension presents challenges for precise patterning on porous substrates. To overcome this limitation, we adopt a high-resolution thermal stamping technique (Fig. 1d), enabling the formation of liquid metal interconnects with a minimum feature size of 50 μm. This approach not only enhances integration density but also ensures robust electrical performance under extreme mechanical deformations.

The stacked layers were tightly bonded to form an integrated multilayer system with stretchability and high permeability (Fig. S4). The thickness of each single-layer device is approximately 0.298 mm (Fig. S5a), while the total thickness of the fully integrated multilayer system reaches 1.669 mm (Fig. S5b). To evaluate the system's mechanical resilience, we subjected the integrated platform to various deformation scenarios, including 30% uniaxial stretching, localized poking, and 30° twisting (Fig. 1e). Meanwhile, we subjected the 3D permeable system to durability testing under 1000 cycles of mechanical stress, including 30% tensile strain and 30° bending conditions (Fig. S6). The system retained its structural integrity and electrical functionality across all conditions, demonstrating exceptional mechanical compliance.

### Engineering Permeable Substrates and High-Resolution Liquid Metal Interconnects

The electronic components in this system are integrated onto highly permeable substrates. The fabrication process for these substrates is detailed in Fig. S7a and the Methods section. Styrene–ethylene–butylene–styrene (SEBS) was selected as the elastomeric material and electrospun into large-scale porous microfiber films (Fig. S7b). To further quantify the fiber uniformity, we performed statistical analysis of fiber diameters based on SEM images (Fig. S7c), which revealed a narrow distribution with an average diameter of 15.94 µm (Fig. S7d), confirming the high uniformity of the electrospun fibers. The choice of SEBS as the base material is particularly significant due to its outstanding elasticity and chemical stability, which are crucial for the durability and reliability of wearable electronic devices.

**Fig. 2:**
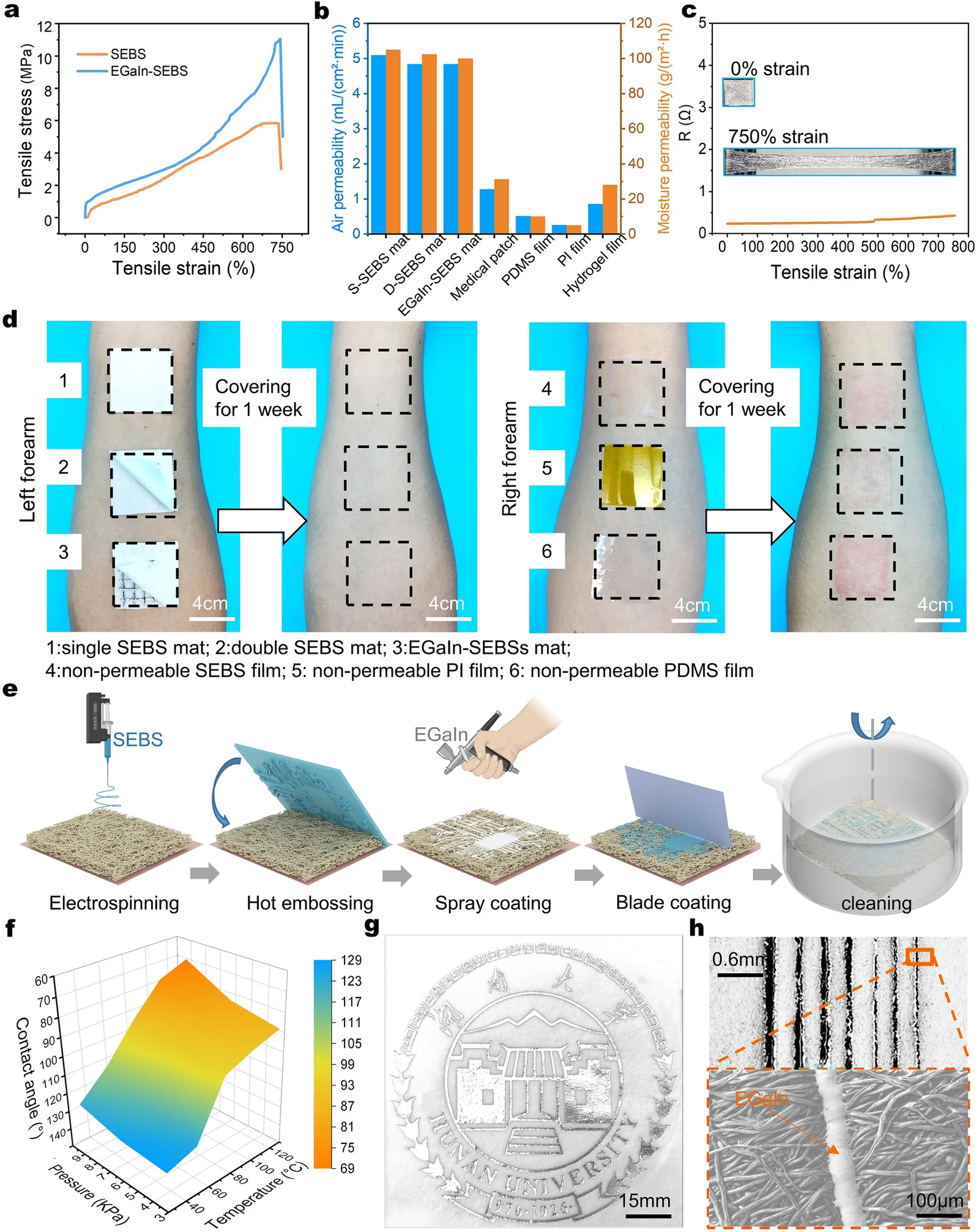
Characterization of permeable substrates and high-resolution patterning of liquid metal interconnects. **a** Stress–strain curves of the electrospun SEBS fiber mat and EGaIn -SEBS fiber mat. **b** Comparison of air and moisture permeability of the S-SEBS, D-SEBS, EGaIn-SEBS medical patch, PDMS film, PI film, and Hydrogel film. **c** Resistance variation of liquid metal-patterned conductive traces on SEBS fiber mats under longitudinal stretching from 0 to 750% strain. **d** Optical images showing the skin irritation results of different materials on the forearms of the volunteer. **e** Schematic diagram of the liquid metal patterning process on SEBS fiber mats. **f** Influence of thermal imprinting conditions (temperature and pressure) on the wettability of SEBS fiber mat, as characterized by contact angle measurements. **g** Optical images of complex liquid metal patterns fabricated on SEBS fiber mat using a thermal imprinting technique. **h** Optical and SEM image of an array of line-shaped liquid metal patterns

Mechanical characterization revealed that the SEBS fiber mat, along with the patterned liquid metal interconnects, exhibits a fracture strain of 750% (Fig. 2a). Moreover, the SEBS fiber mat exhibited excellent long-term cyclic tensile stability at 500% strain (Fig. S8). Notably, the Young’s moduli of these materials closely match that of human skin, ensuring comfort and mechanical resilience in wearable applications. Furthermore, we evaluated the air and moisture permeability of single-layer SEBS fiber mat (S-SEBS), double-layer SEBS fiber mat (D-SEBS), and SEBS fiber mat coated with conducting liquid metal (EGaIn-SEBS) (Fig. 2b). The results indicate that air permeability remains consistent across different layer conFig. urations and is significantly higher than conventional flexible substrates such as medical patch, PDMS films PI films and Hydrogel films. The air permeability and moisture permeability of these porous substrates, combined with liquid metal conductors, are exceptionally high (5.09295 mL cm^−2^ min^−1^ and 105 g m^−2^ h^−1^ = 2520 g m^−2^ day^−1^, respectively), effectively accommodating human perspiration. Electrical characterization demonstrated that the patterned liquid metal on SEBS fiber mat maintained stable electrical resistance under 750% tensile strain (Fig. 2c). Furthermore, to evaluate the long-term durability of the patterned liquid metal on SEBS fiber mat, cyclic tensile testing up to 32,500 cycles at 100% strain was conducted. The results (Fig. S9) show negligible resistance drift, confirming excellent electrical stability of the conductive pathways under prolonged deformation.

Biocompatibility is of paramount importance for wearable and on-skin applications. EGaIn-SEBS and SEBS were affirmed to possess low cytotoxicity by an in vitro study using L-929 cells as the model cell. Bright-field and fluorescent live/dead staining images showed a regular cell morphology and scarce dead cells in all groups except for the positive control group, which experienced severe cell death (Fig. S10). Additionally, to validate skin compatibility, a one-week wearability test was conducted using eight samples adhered to a volunteer’s forearm (Fig. 2d). The results confirmed that S-SEBS, D-SEBS, and EGaIn-SEBS mats caused no adverse skin reactions, whereas conventional non-permeable substrates such as SEBS films, PI films, and PDMS films resulted in significant skin erythema. Meanwhile, infrared thermal imaging (Fig. S11) demonstrated that S-SEBS, D-SEBS, and EGaIn-SEBS mats exhibited superior thermal comfort, with virtually no temperature rise observed on the skin surface after 15 min of running, whereas the corresponding skin area of SEBS films, PI films and PDMS films showed an average temperature increase of 3 °C. These findings highlight the potential of permeable SEBS fiber mat for applications where permeability is paramount, such as interactive robotics and electronic skin prosthetics.

Patterning liquid metal onto SEBS fiber mat presents a significant challenge due to the high surface tension of the metal, which hinders uniform deposition on rough surfaces. To address this, we developed a fabrication process based on thermal imprinting technology to pattern liquid metal onto SEBS fiber mat (Fig. 2e). The SEBS fiber mat, fabricated via electrospinning, inherently possesses a rough and porous surface. By applying localized heat and pressure, we modulated the surface energy of designated regions, increasing their wettability to liquid metal while leaving non-selected regions unchanged. This differential wettability enables high-resolution patterning, a capability often limited in conventional liquid metal-based electronics.

Following this surface modification, liquid metal was ultrasonically dispersed in ethanol and uniformly sprayed onto the substrate using a spray gun. A subsequent scraping process selectively removed excess metal, forming well-defined conductive pathways. To empirically validate the effectiveness of our thermal imprinting process, we conducted contact angle measurements (Fig. S12). The results demonstrate that with optimized parameters (temperature: 125 °C, pressure: 9 kPa), the contact angle decreased from 129.3° to 68.7°, significantly enhancing liquid metal adhesion (Fig. 2f). To further evaluate long-term reliability, we conducted an accelerated aging test by immersing thermally imprinted liquid metal patterns encapsulated within SEBS in artificial sweat (0.9% NaCl) for 7 days. The resistance was continuously monitored with an LCR meter and remained highly stable with negligible fluctuation (Fig. S13), confirming excellent resistance to oxidation, migration, and adhesion degradation under physiological conditions. Building upon this, we successfully fabricated various liquid metal-patterned structures, including straight lines, curved traces, and complex arbitrary geometries (Figs. 2g and S14). The embossing templates used in this process were manufactured via 3D printing technology (Fig. S15). Currently constrained by the fabrication precision of the templates, the achievable pattern resolution reaches 50 μm (Fig. 2h). This resolution surpasses most existing fast and cost-effective liquid metal patterning techniques on porous substrates, with the exception of photolithography, which requires costly equipment and materials [11, 28]. To further contextualize the advantages of our platform, we provide a quantitative comparison with representative state-of-the-art stretchable and breathable electronic systems in Table S1. The comparison highlights that our system achieves a rare combination of high integration density (7.9 cm^−2^), ultrahigh permeability (> 2520 g m^−2^ day^−1^), sub-50 μm patterning resolution, and robust cycling performance under 32,500 strain cycles, collectively underscoring its novelty and performance advantages.

### Strain-Isolated Vertical Interconnects for Robust Multilayer Integration

**Fig. 3:**
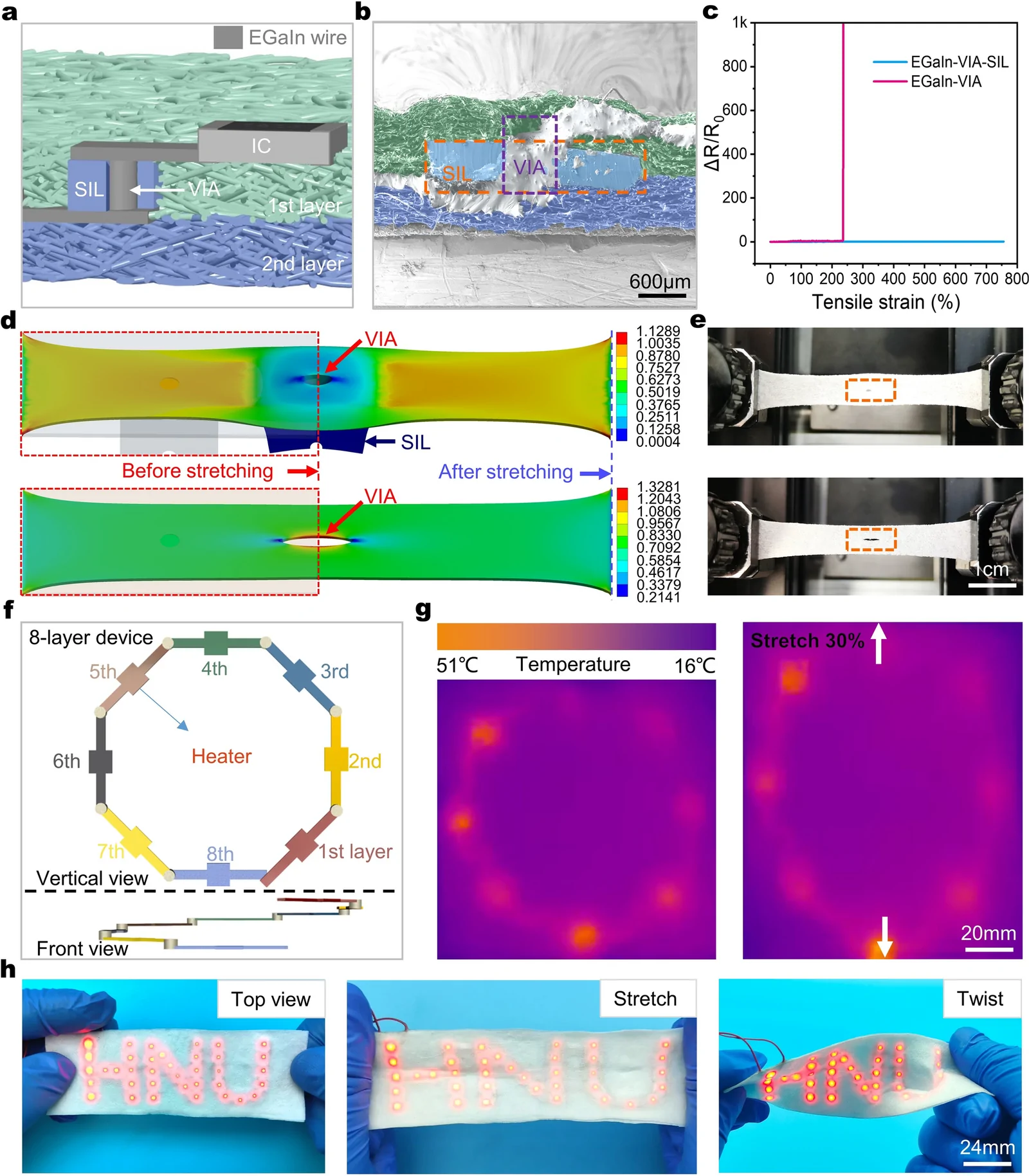
Design of SIL and characterization of VIA stretching performance. **a** Schematic illustration of a 3D electrical interconnect system. VIAs serve as interlayer electrical pathways, while the SIL protects them from mechanical strain during deformation. **b** Cross-sectional SEM images showing a 3D electrical interconnect system. **c** Electrical resistance variation of VIAs with (EGaIn-VIA-SIL) and without (EGaIn-VIA) SIL protection during stretching. **d** Finite element simulation illustrating strain distribution in VIAs with and without SIL protection under 100% tensile strain. The red dashed line represents the unstretched reference state. **e** Experimental validation of VIA deformation with and without SIL protection under 100% tensile strain. The red dashed box highlights the physical deformation of the VIA structure. **f** Schematic diagram of the structure of an 8-layer stretchable heater. **g** (Left) Top view thermal image of the eight-layer stretchable heater. (Right) Thermal image of the heater under 30% uniaxial strain. **h** Optical micrographs of the 3D integrated LED array system with SIL protection, shown in three states: independent (top), stretched to 100% strain (middle), and twisted 90 degrees (bottom)

Vertical interconnect accesses (VIAs) are essential for enabling interlayer electrical connections in three-dimensional integrated systems, enhancing both integration density and functional complexity. However, when employing permeable SEBS fiber mat as substrates, substantial VIA deformation can occur during stretching (Fig. S16), potentially disrupting interlayer electrical pathways. To address this challenge, we designed a strain isolators (SIL), as illustrated in Fig. 3a. The SIL effectively maintains the structural integrity of the VIAs, ensuring stable electrical performance even under significant tensile deformation. Figure 3b shows the corresponding SEM image.

The SIL must exhibit sufficient mechanical stability to protect the VIA while preserving the overall flexibility of the multilayer electronic system, allowing it to conform to the uneven surfaces of human skin. Figure S17 presents a three-dimensional schematic and scanning electron microscopy (SEM) images of the SIL structure. In our design, VIAs facilitate vertical electrical connections between layers, while horizontal interconnect accesses (HIAs) provide in-plane lateral connections within individual layers. SEBS, the same elastomeric material used for the substrate, was chosen to fabricate the SIL (See Sect. 2.4 in the Methods part), ensuring robust adhesion between the SIL and the underlying substrate. SIL plays a central role in ensuring the mechanical and electrical reliability of vertical interconnects under repeated large deformations. Mechanically, the SIL functions as a soft buffer that redistributes strain away from the VIAs.

To validate the effectiveness of SIL-protected VIAs (EGaIn-VIA-SIL) compared to unprotected VIAs (EGaIn-VIA), we conducted electrical resistance tests (Fig. 3c). The results demonstrated that EGaIn-VIA-SIL maintained low and stable resistance under strains of up to 750%, whereas EGaIn-VIA lost electrical connectivity at 250% strain. To further investigate the impact of SILs on VIA deformation, we performed finite element simulations (FEA) under 100% tensile strain (Fig. 3d). The upper panel of Fig. 3d shows that in SIL-protected VIAs, maximum strain (1.1289 mm mm^−1^) is redistributed externally by the SIL, preventing significant deformation within the VIA. SIL effectively reduces stress concentration by modulus mismatch buffering and provides a smooth transition between the VIAs and the porous nanofiber substrate. This design enables strain redistribution, where deformation is absorbed predominantly by the SIL itself rather than the via interface. In contrast, unprotected VIAs (Bottom panel) experience maximum strain (1.3281 mm mm^−1^) concentrated within the interconnect, leading to substantial deformation and an increased risk of failure. To validate the finite element simulation results (Fig. 3d), we conducted a comparative tensile experiment. Under 100% tensile strain, the top panel of Fig. 3e shows that the VIA protected by the SIL remained structurally intact, whereas the unprotected VIA in the control group (bottom panel) exhibited significant structural deformation. To further validate long-term durability, cyclic strain tests were conducted up to 32,500 cycles at 100% strain, and the results (Fig. S18) showed minimal resistance drift, confirming the robustness of the SIL design for repeated deformation.

The utilization of the SIL has proven to be an effective strategy for preventing excessive deformation in interlayer VIAs, thereby preserving electrical connectivity in multilayer stretchable electronic systems. To demonstrate the scalability of this approach, we fabricated an 8-layer stretchable heater, as illustrated in Fig. 3f. The heater comprises multiple layers interconnected through SIL-reinforced VIAs, with heating elements positioned at the midpoint of each layer's conductive pathway. Thermal images of the heater in operation (Fig. 3g, left) confirm proper electrical insulation between layers, with no detectable short-circuit occurrence. Furthermore, thermal imaging under 30% uniaxial strain (Fig. 3g, right) demonstrates stable performance during mechanical deformation.

To further validate the robustness of SIL-integrated VIAs, we developed a 3D integrated LED array (Fig. 3h). Fabrication details are provided in the Methods section. This LED array maintained high mechanical stability under both stretching and bending conditions (Movie S2). Additionally, we immersed the system in stirred water for an extended period (Movie S3), confirming its continued functionality despite exposure to dynamic liquid environments. These results highlight the effectiveness of our SIL design in enhancing the mechanical reliability of VIAs within highly integrated, stretchable 3D electronic systems.

### Multichannel Data Acquisition for Real-Time Gesture Recognition

**Fig. 4:**
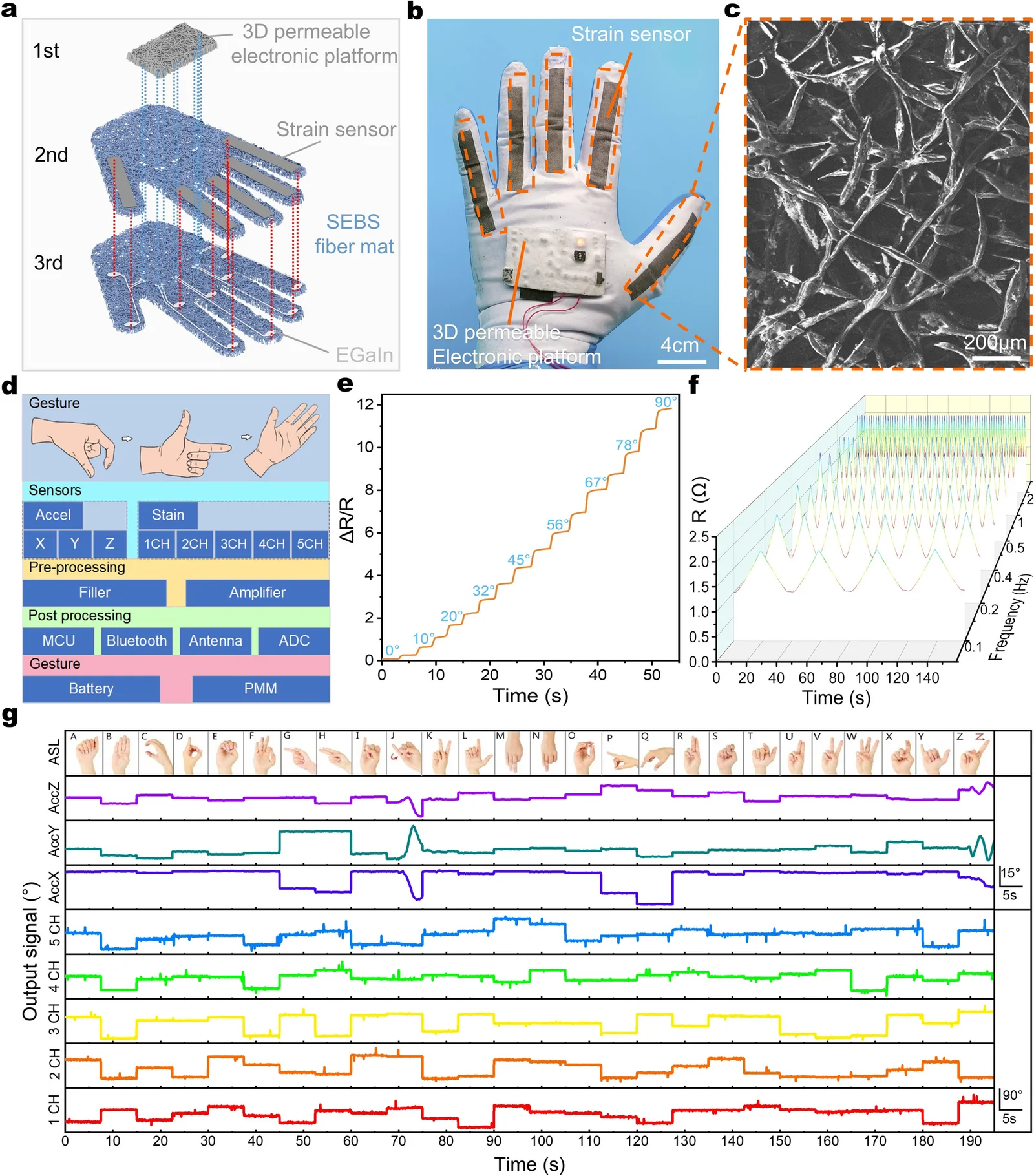
Characteristics of the flexible, stretchable, and permeable strain sensor and its signal acquisition. **a** Exploded schematic of the gesture acquisition system. **b** Optical image of the complete gesture acquisition system. **c** Scanning electron microscopy (SEM) image of the strain sensor. **d** Schematic diagram depicting the structural composition of the gesture acquisition circuit. **e** Resistance response of the gesture acquisition system to varying finger curvatures. **f** Electrical response characteristics of the strain sensor under 100% tensile strain at different frequencies. **g** Real-time signal variations from five strain sensors and a three-axis accelerometer, corresponding to different hand movements and gestures

To demonstrate the applications of the 3D integrated permeable system in wearable electronic scenarios, we developed a stretchable and permeable strain sensor and integrated the two components (Fig. 4b) for real-time human gesture recognition. An exploded schematic of the glove is provided in Fig. 4a. The back of the glove features an embedded 3D integrated system circuit, with its internal structure detailed in Fig. 4d. (The system circuit schematic is provided in Fig. S19). The sensing components include a three-axis accelerometer and five-channel strain sensors, enabling real-time gesture recognition through signal processing. Five strain sensors are positioned on the back of the glove’s fingers.

Figure S20 shows the fabrication process of stretchable and breathable strain sensors (Fig. S21a). Scanning electron microscopy (SEM) images (Fig. 4c) show that the sensors exhibit a porous mesh structure, with a liquid metal coating serving as the active sensing material. The porous structure demonstrates exceptional air permeability and moisture permeability (Fig. S22b), with performance parameters significantly superior to conventional medical tapes. Meanwhile, the strain sensor also exhibits excellent stretchability (Fig. S21b), which markedly enhances wearing comfort and reduces skin irritation risks. These characteristics are critically important for long-term wearable applications.

The conductivity and sensitivity of the strain sensors are strongly influenced by the quantity of liquid metal applied as the conductive layer. As illustrated in Fig. S22a, three experimental groups were tested, using 0.5, 0.7, and 0.9 g of liquid metal, which was uniformly coated onto the SEBS fiber mat through multiple rolling processes. This process formed a conductive composite comprising liquid metal and its oxides. Under tensile deformation, the electrical resistance of the fiber increased significantly. The results indicated that the strain sensor with 0.5 g of liquid metal (0.5EGaIn-SEBS) exhibited high sensitivity (GF = 4.423) under large strain conditions.

To further evaluate sensor performance, we examined its resistance response under a maximum strain of 100% across varying stretching frequencies (Fig. 4f). The results show that the sensor can adapt to various frequency responses. Meanwhile, the sensor accurately quantified finger bending degrees through resistance changes without noticeable signal lag. As shown in Fig. 4e, resistance increased proportionally with bending angle, demonstrating the sensor’s capability to precisely track finger motions. Furthermore, even after 2000 stretching cycles, the sensor maintained a stable resistance variation (Fig. S22c), highlighting its durability and long-term reliability. A slight resistance increase was observed after extended cycling, which we attribute to the redistribution of liquid metal within the elastomer matrix and the accumulation of surface oxides during deformation (Fig. S23). This mechanism accounts for the gradual resistance rise while confirming that the overall conductivity and functionality remain highly stable.

The strain sensor employs the same architectural principles as the permeable 3D circuit, reinforcing the flexibility and comfort of wearable devices while enhancing their functional capabilities. This integration allows for accurate gesture recognition and motion tracking. Figure 4g presents the eight-channel signals acquired from the sensor array, corresponding to the 26 letters of the English alphabet, demonstrating the system’s efficacy in gesture-based input applications.

### Sign Language Interpretation and Interactive Robotics

To showcase the capabilities of 3D permeable integrated circuits and sensors, we developed a wireless system designed for real-time, high-precision monitoring and recognition of sign language. This innovation facilitates sign language interpretation and enhances communication for individuals with hearing and speech impairments.

**Fig. 5:**
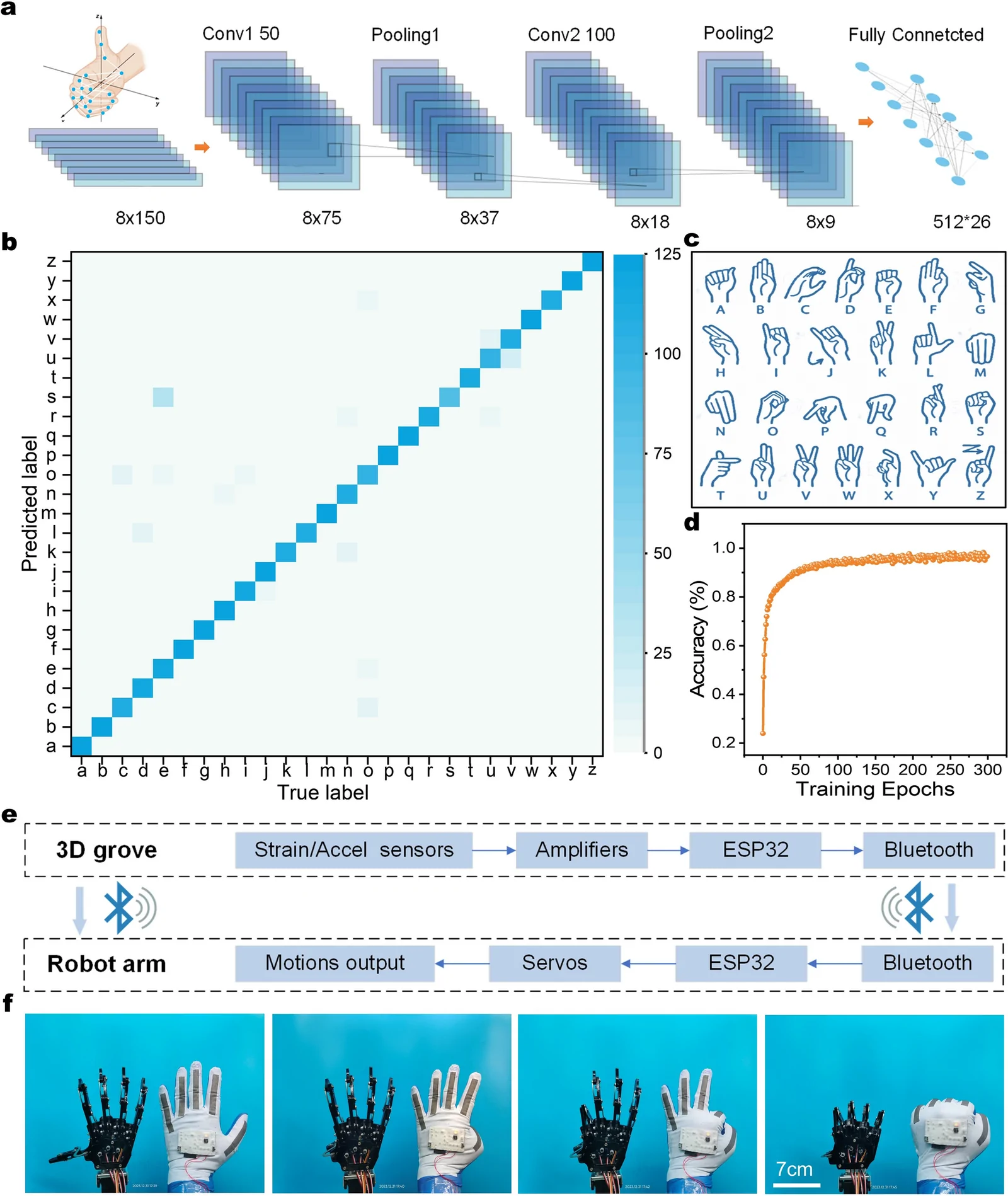
Sign language recognition and interactive robotic arm control. **a** Architecture of the convolutional neural network (CNN) model used for gesture recognition. **b** Confusion matrix of CNN-based recognition for the 26 English alphabet letters. **c** Visual representation of hand gestures corresponding to the 26 English alphabet letters. **d** CNN recognition accuracy of the 26 English alphabet letters. **e** System-level block diagram of the robot controlling system. **f** Demonstration of the wireless glove-based system controlling a robotic hand in real-time, accurately translating human gestures into robotic movements

The core recognition module of the system employs a convolutional neural network (CNN), as illustrated in Fig. 5a. The model architecture consists of two convolutional layers, two pooling layers, and two fully connected layers, with detailed parameters provided in Table S2. The system acquires 26 English alphabet gesture data through an 8-channel signal acquisition device (Fig. 5c). A total of 2,600 samples were collected (20 volunteers × 26 gestures × 5 repetitions), with 100 samples corresponding to each letter gesture. A stratified random partitioning strategy was adopted: 1,820 samples (70%) for training set, 390 samples (15%) for validation set, and 390 samples (15%) for test set to ensure data distribution consistency. After standardized preprocessing, the data were input into a CNN model comprising dual convolutional-pooling layers and fully connected layers, which converged after 300 training epochs. Test set evaluation (Fig. 5d) demonstrated a classification accuracy of 98%. Confusion matrix analysis (Fig. 5b) revealed consistently high recognition rates for all gestures, with an average diagonal recognition rate of 96.8%. Real-time performance tests confirmed that the system completes gesture recognition within 100 ms, meeting real-time interaction requirements. To further underscore the unique advantages of our system, we have included a systematic comparison with representative commercial and academic solutions (Table S3). The results reveal that our platform combines outstanding air permeability (5.09295 mL cm^−2^ min^−1^)), excellent moisture permeability (2520 g m^−2^ day^−1^), high stretchability (750%), high recognition accuracy (98%), and good biocompatibility, offering superior overall performance for long-term wearable applications.

As a proof of concept, we implemented a wearable, wireless gesture acquisition and recognition system optimized for long-term use. This system was applied to the real-time remote control of a robotic manipulator with six degrees of freedom. The overall system architecture is depicted in Fig. 5e, illustrating signal transduction, modulation, processing, and wireless transmission pathways. Figure 5f shows the robotic hand replicating human gestures, where the intelligent fabric glove accurately recognizes random gestures and various finger bending angles, seamlessly triggering corresponding robotic actions (Movie S4). This demonstration highlights the potential of smart wearables in human–machine interaction, marking a significant step toward advanced robotic control through intuitive, gesture-based inputs.

## Conclusions

In this work, we have demonstrated a wearable electronic system through a three-dimensional integration strategy that fundamentally addresses the demands of physiological comfort and high-density functionality. By leveraging electrospun nanoporous SEBS fiber mat with precision thermal imprinting (50 μm resolution) of liquid metal interconnections, we achieved a mechanically robust yet highly flexible electronic architecture. Furthermore, the introduction of a strain isolators (SIL) ensures the interfacial reliability of vertical interconnects under large deformations, enabling stable multilayer integration. To showcase its practical applications, we developed a wireless gesture recognition textile glove, which effectively translates complex hand gestures into electronic signals, demonstrating its potential in sign language recognition and robotic control. Despite these advances, we acknowledge that the current fabrication process still relies on thermal imprinting and spray coating, which may lead to yield variations due to alignment tolerances and fiber porosity fluctuations. Although thermal imprinting is scalable in principle, further efforts toward integrating roll-to-roll compatible imprinting and automated assembly are ongoing to enhance reproducibility and industrial scalability. The platform’s superior mechanical compliance, permeability, and durability position it as a transformative technology for next-generation wearable electronics, interactive robotics, and human–computer interfaces.

## Supplementary Information

Below is the link to the electronic supplementary material.

Supplementary file1 (MP4 1412 KB)

Supplementary file2 (MP4 534 KB)

Supplementary file3 (MP4 1419 KB)

Supplementary file4 (MP4 2562 KB)

Supplementary file5 (DOCX 31471 KB)
