# Supplementary material for: A Highly Permeable and Three-Dimensional Integrated Electronic System for Wearable Human–Robot Interaction
Source: Nanomicro Lett. 2026 Jan 3;18:128. doi: 10.1007/s40820-025-01974-z (PMC12759031; doi:10.1007/s40820-025-01974-z)
Supplement: Supplementary file 5 — Supplementary file5 (DOCX 31471 KB) [file 40820_2025_1974_MOESM5_ESM.docx]

Supporting Information for

**A Highly Permeable and Three-Dimensional Integrated Electronic System for Wearable Human-Robot Interaction**

Wenqiang Wang^1^, Zebang Luo^1^, Xingge Yu^1^, Xiaojia Yin^1^, Li Xiang^1*^, Anlian Pan^1,2*^

^1^ Key Laboratory for Micro-Nano Physics and Technology of Hunan Province, State Key Laboratory of Chemo/Biosensing and Chemometrics, Hunan Institute of Optoelectronic Integration, College of Materials Science and Engineering, Hunan University, Changsha, 410082, P. R. China

^2^ School of Physics and Electronics, Hunan Normal University, Changsha 410081, P. R. China

*Corresponding authors. E-mail: [xiangli93@hnu.edu.cn](mailto:xiangli93@hnu.edu.cn) (Li Xiang); [anlian.pan@hnu.edu.cn](mailto:anlian.pan@hnu.edu.cn) (Anlian Pan)

**Note S1 Fabrication of LED Array**

The LED array was fabricated using a multilayer stacking structure (Fig. S26), consisting of a top encapsulation layer, the LED array body, and a bottom encapsulation and interconnection layer. The top encapsulation layer is a porous SEBS fibre mat, serving as the outermost protective layer for the circuit and providing waterproofing. The LED array body, consisting of conductors and LED lights, forms the core structure. The conductors were patterned on the SEBS fibre mat using liquid metal to form an HNU pattern, followed by sequential soldering of the LEDs to the patterned conductors. The correct alignment of the LED polarity and conductors was ensured during soldering for proper circuit operation. The bottom encapsulation and interconnection layer, made from the same material as the top encapsulation, connects the LED array and provides bottom packaging, with VIA reinforced using SIL.

**Note S2 Preparation of Stretchable and Permeable Liquid Metal-Based Strain Sensors**

Stretchable and permeable liquid metal-based strain sensors were fabricated as follows (Fig. S20). A mixture of liquid metal and ethanol (2:5 mass ratio) was prepared and subjected to ultrasonic treatment (10W) for 5 minutes to disperse the liquid metal into fine droplets with uniform particle size distribution. The SEBS fibre mat was cleaned using a plasma cleaner to activate its surface and improve adhesion to the liquid metal. The liquid metal was then sprayed onto the pre-treated SEBS fibre mat using a spray device. A rolling press machine applied 10 N of pressure for 10 reciprocating cycles to ensure tight contact between the conductive liquid metal and the SEBS fibre mat, thereby achieving optimal sensor performance.

**Note S3 Fabrication of Fabric Gloves**

The fabric gloves were constructed with a three-layer structure (Fig. S4a). The first layer, located on the outermost part of the glove, consists of a 3D permeable electronic platform, which is used to collect and process signals and wirelessly transmit them to a host computer. The second layer, situated in the middle, is composed of five strain sensors positioned on a SEBS fibre mat, designed to detect the bending angles of the fingers. The third layer, at the bottom, comprises interconnected conductive wires patterned using liquid metal on the SEBS fibre mat. These wires connect the strain sensors to the 3D electronic platform, enabling signal transmission for gesture detection and recognition.

**Note S4 Investigation of the cell cytotoxicity of EGaIn-SEBS**

In vitro cytotoxicity testing of as-prepared SEBS and EGaIn-SEBS samples was performed using L-929 cells. Briefly, 6×10⁴ L-929 cells were seeded per well in a 24-well plate with 1 ml of complete culture medium. After 24 hours of incubation, during which cell confluence and morphology were verified, the existing medium was discarded and replaced with 1 ml of fresh complete medium. Sterile SEBS or EGaIn-SEBS fiber mats (0.2 cm²) were then directly placed into the wells to assess material cytotoxicity. Control groups included fresh complete medium (blank control), 0.2 cm² medical absorbent gauze (negative control), and a 20% DMSO/complete medium mixture (positive control). All samples were cultured at 37°C in a 5% CO₂ incubator. After 24 hours of incaution, cell viability and proliferation were quantitatively evaluated using live/dead staining assays.

**Supplementary Figures and Tables**


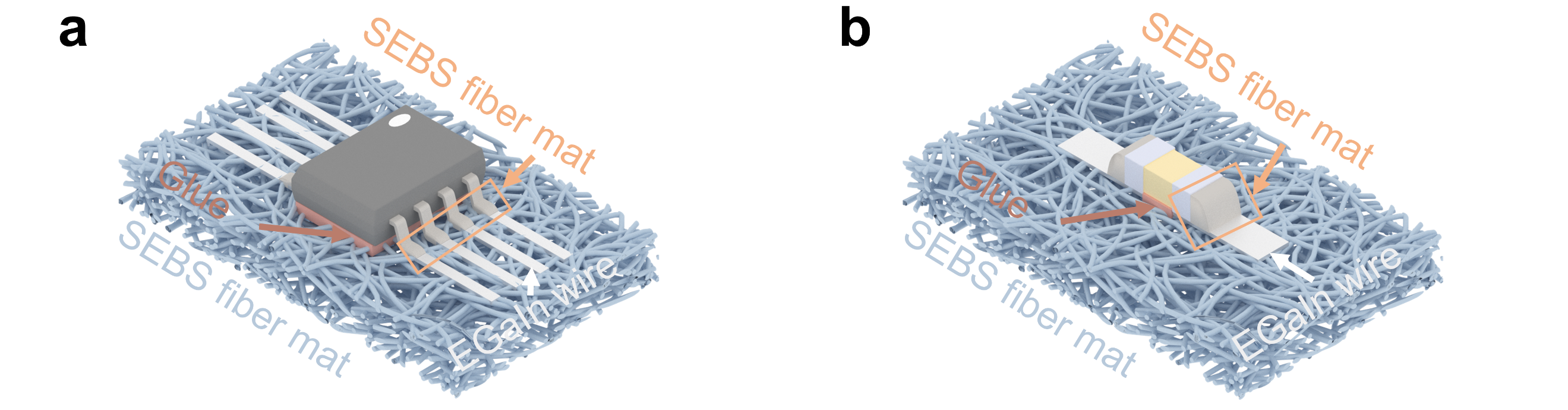


**Fig. S1** Fixation methods for electrical components on circuits. **a** Chip with leads. **b** Chip without leads


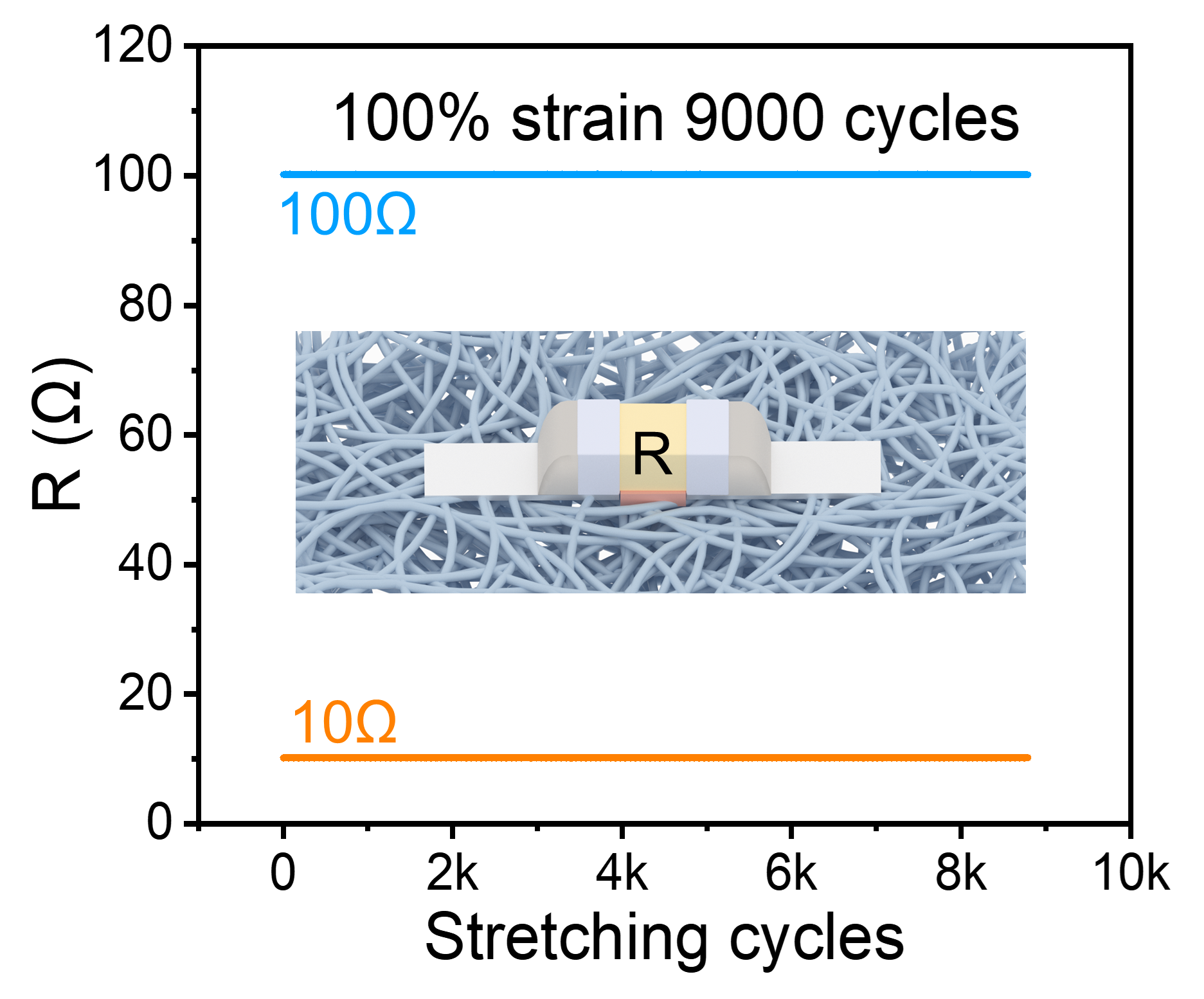


**Fig. S2** An array of stretchable micro-resistor-based circuits exhibiting excellent resistance stability under 100% strain over 9000 cycles


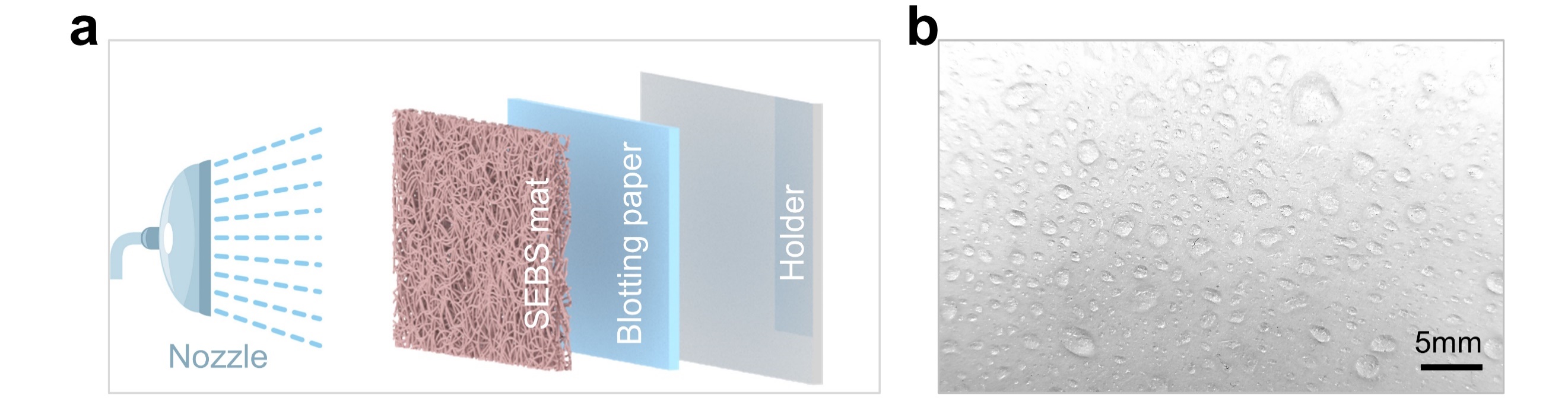


**Fig. S3** Waterproof performance characterizations of SEBS mat. **a** Schematic illustration of the standard rain test (AATCC Test Method 35–2006). **b** Optical photographs demonstrate the surface condition of the SEBS mat after water exposure testing


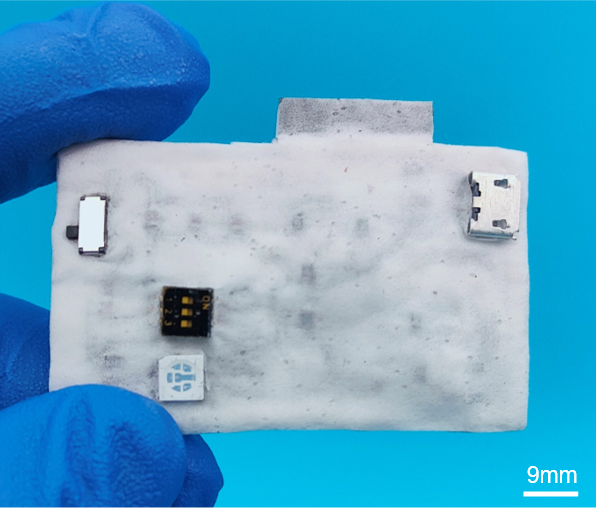


**Fig. S4** Optical images of the 3D permeable electronic platform


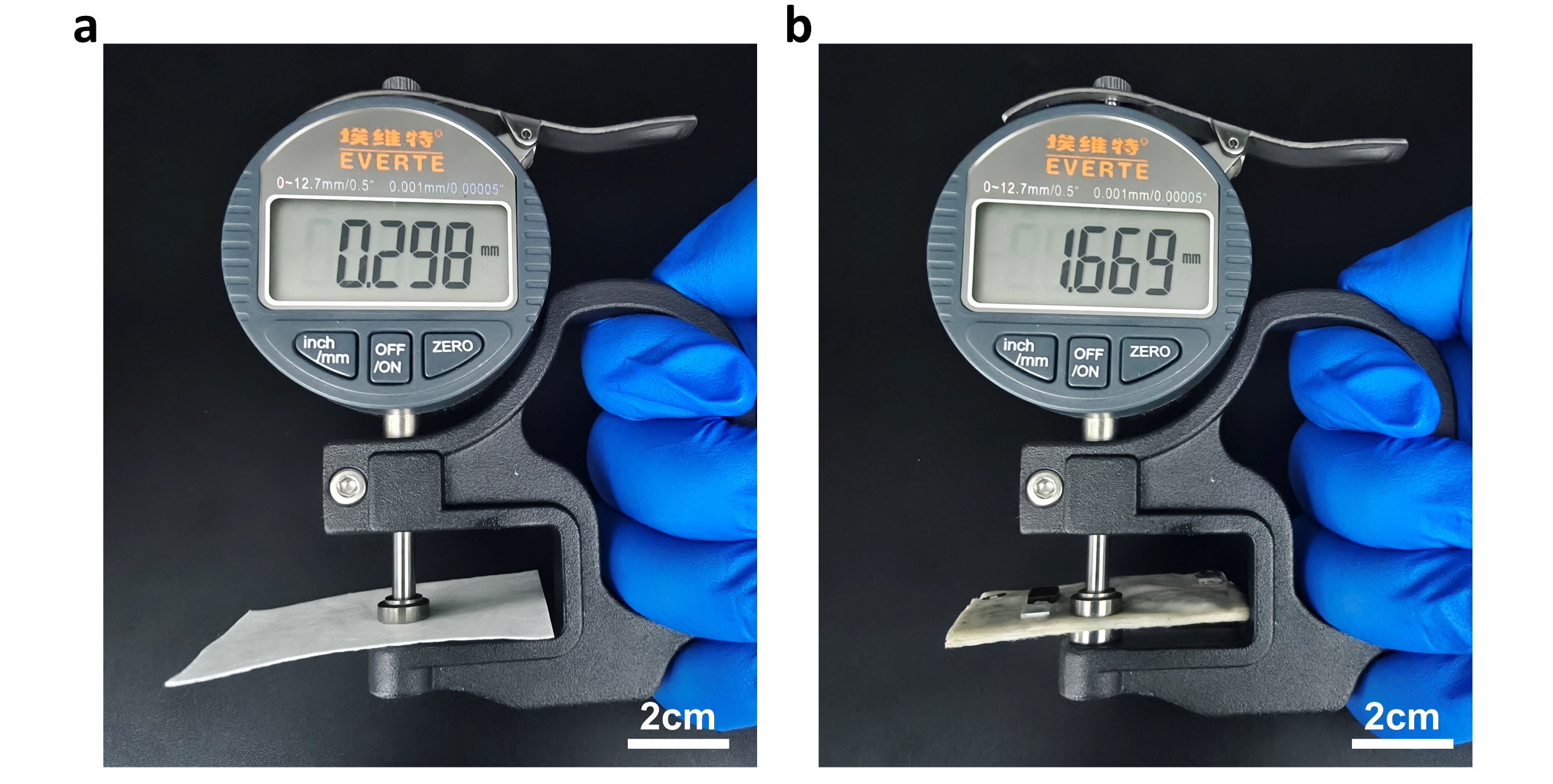


**Fig. S5 a** Digital images showing the overall thickness of the single-layer device. **b** Digital images showing the overall thickness of the multi-layer device


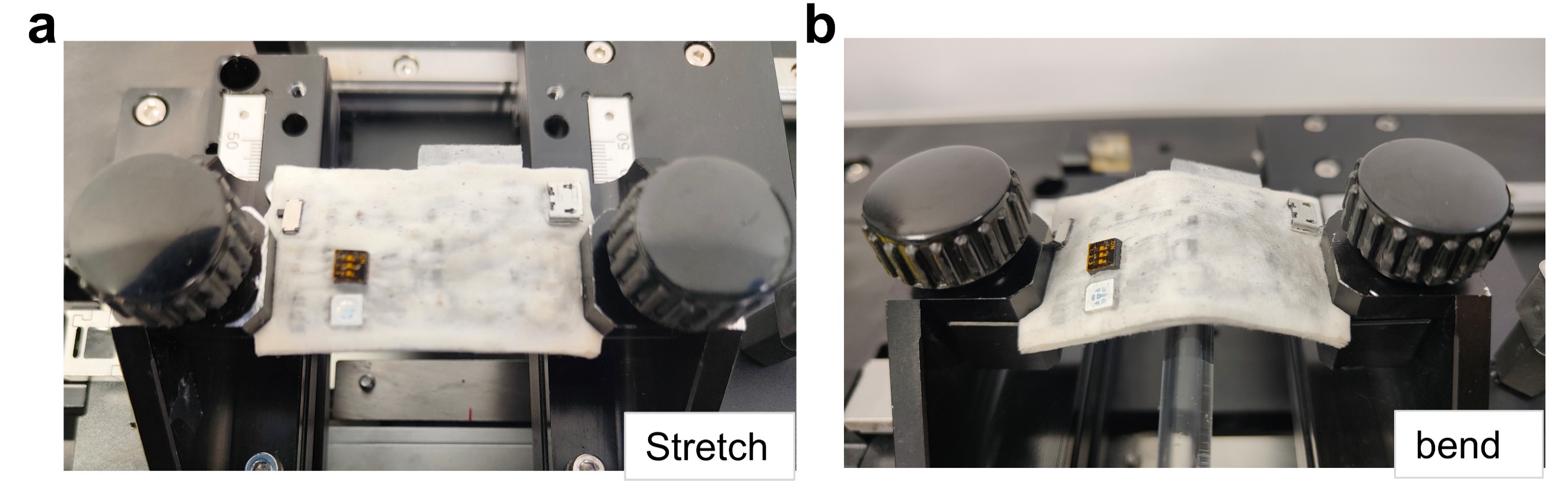


**Fig. S6** The 3D permeable system maintains mechanical compliance and durability under 1000 cycles of mechanical stress. **a** Cyclic stretching at 30% strain. **b** Cyclic bending at 30°


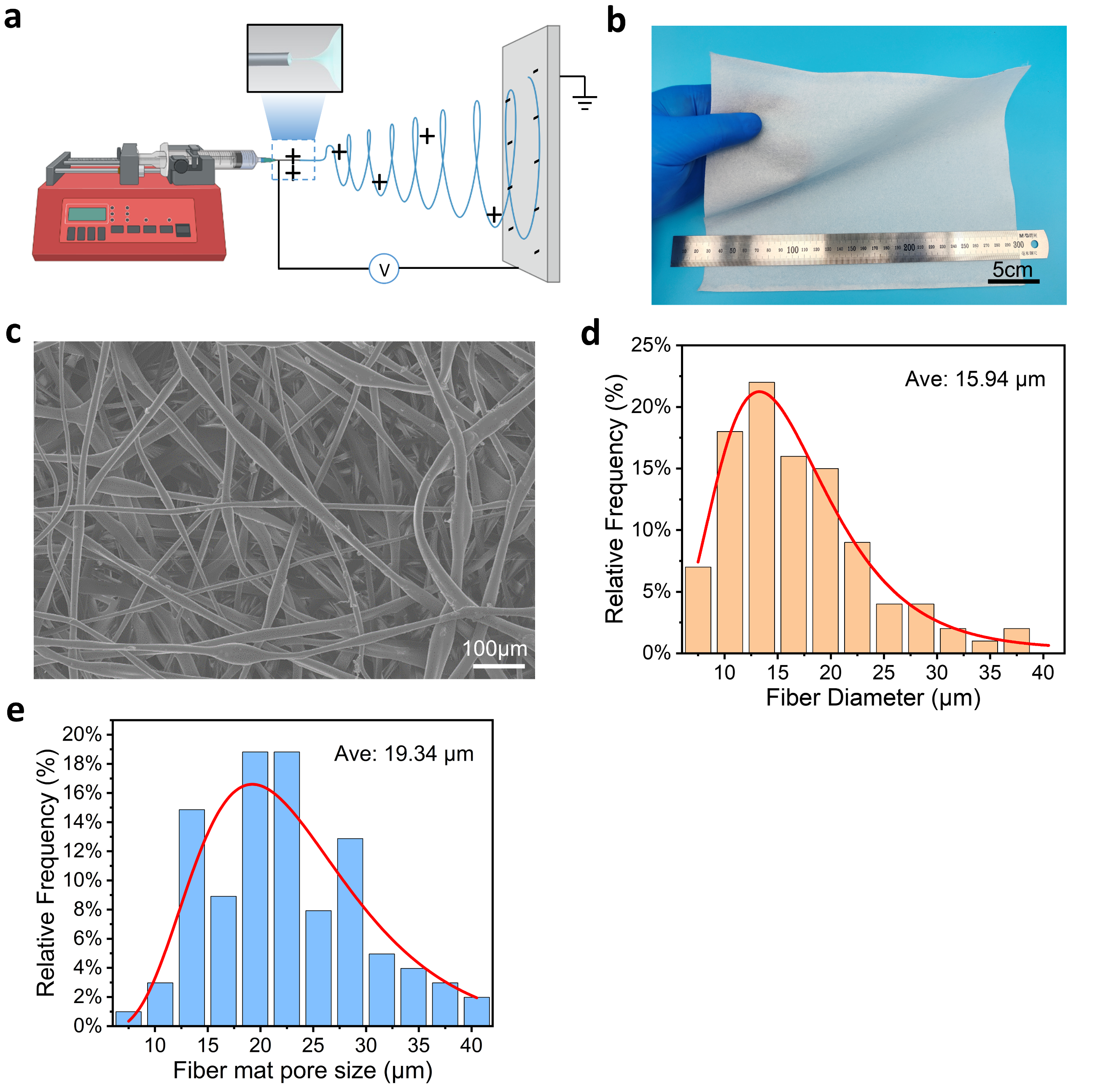


**Fig. S7** Preparation and Characterization of Electrospun Films. **a** Electrospinning process diagram. **b** Optical images of SEBS fibre mat. **c** SEM images of SEBS fibre mat. **d** SEM-based diameter distribution analysis of SEBS electrospun fibers. **e** SEM-based pore size distribution analysis of SEBS fiber mat


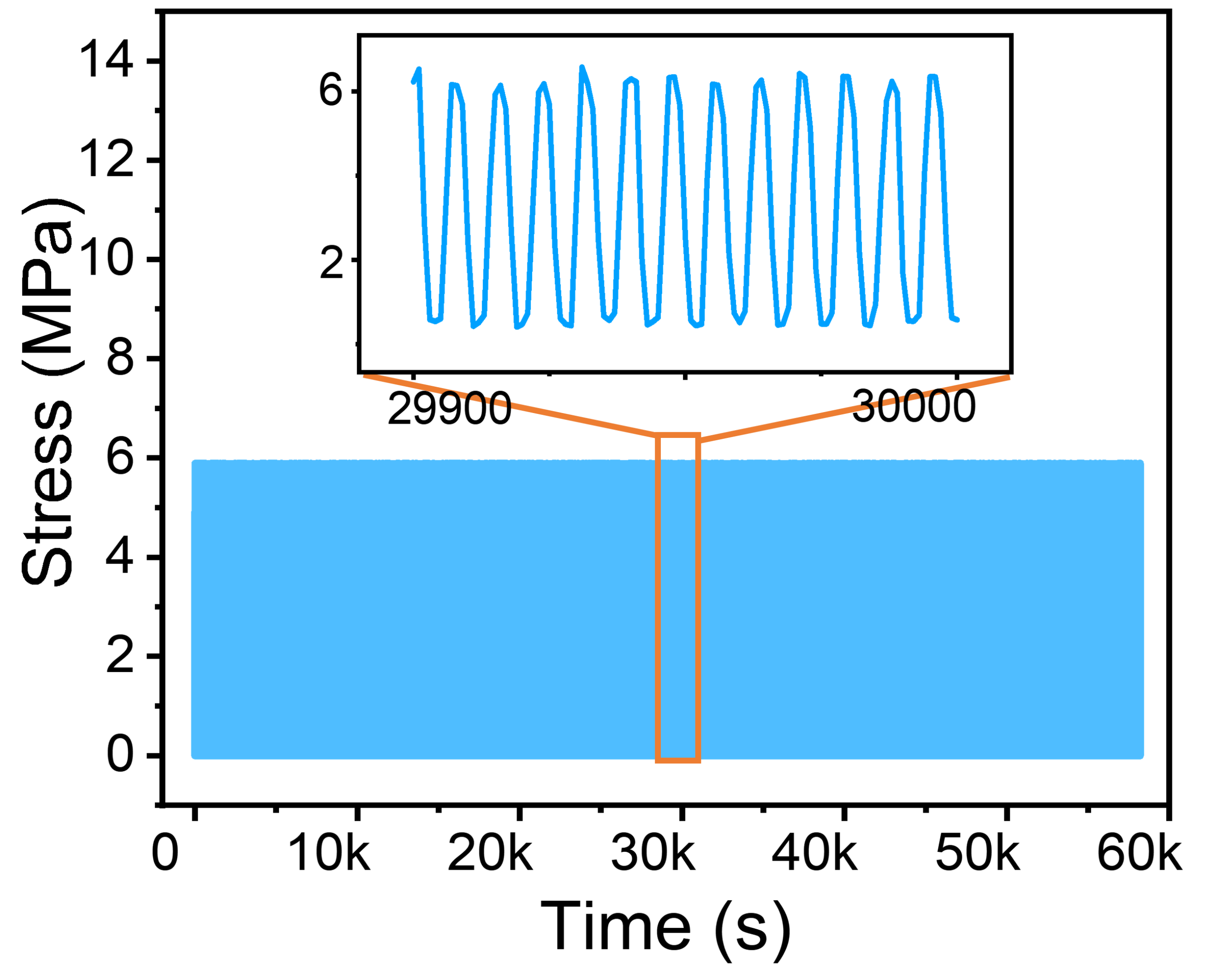


**Fig. S8** Long-term cyclic tensile behavior of a SEBS fibre mat under 500% strain


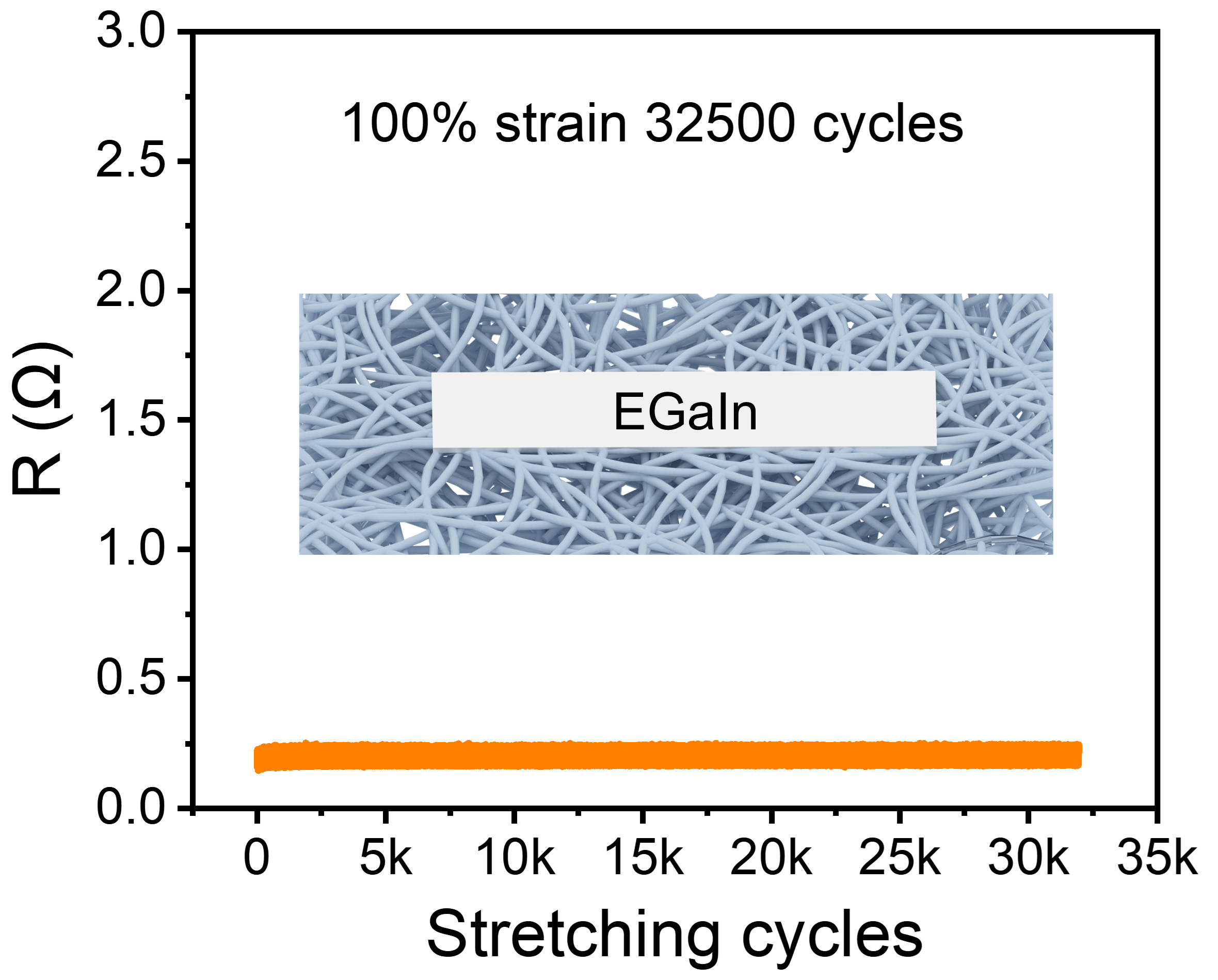


**Fig. S9** Cyclic stability testing of the conductive layer on SEBS under 100% tensile strain


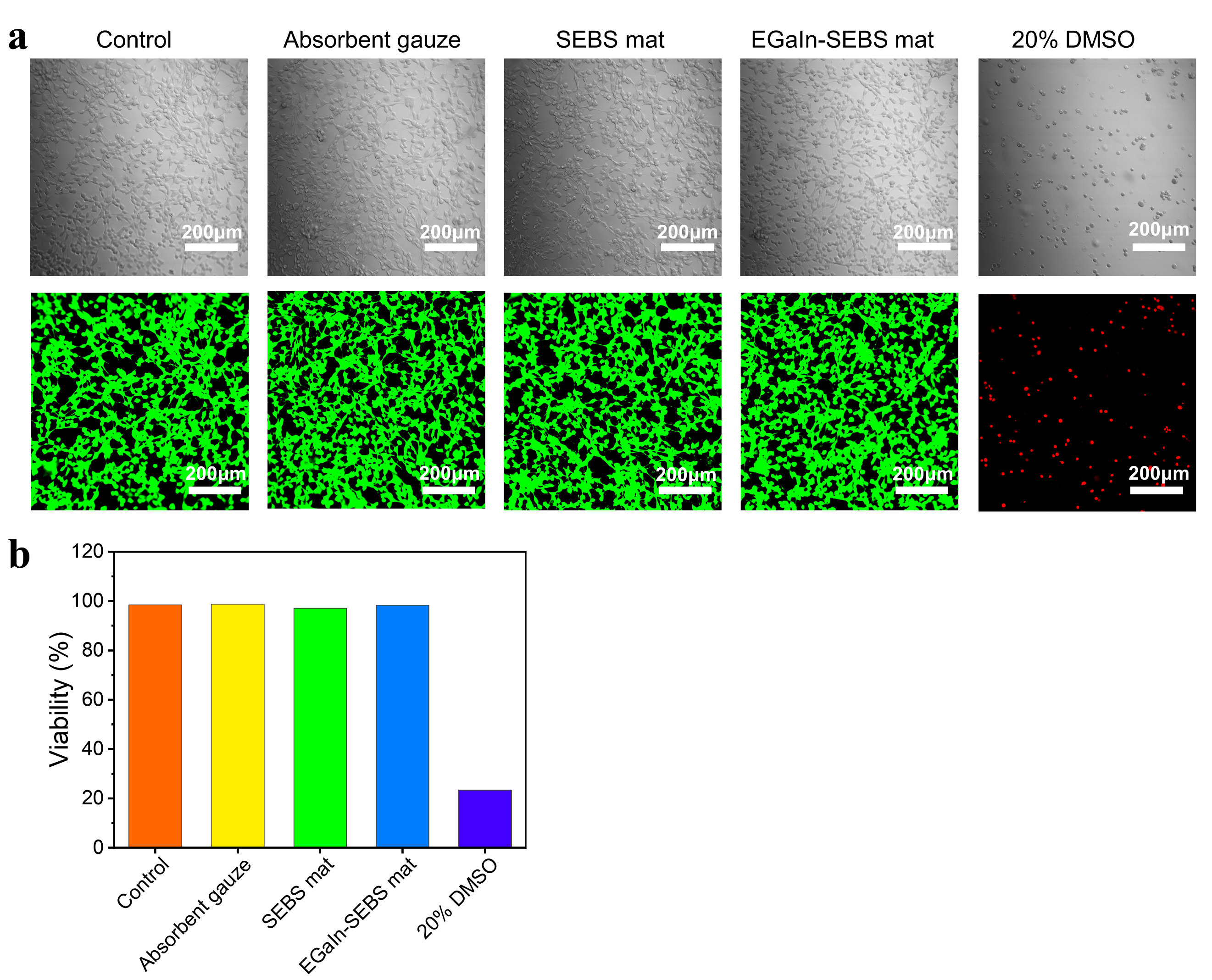


**Fig. S10 a** Bright-field and fluorescent images of cells cultured in the incubation medium with the control sample, absorbent gauze, SEBS mat, EGaIn-SEBS and 20% DMSO. **b** Quantification of L-929 cell viability in different incubation groups


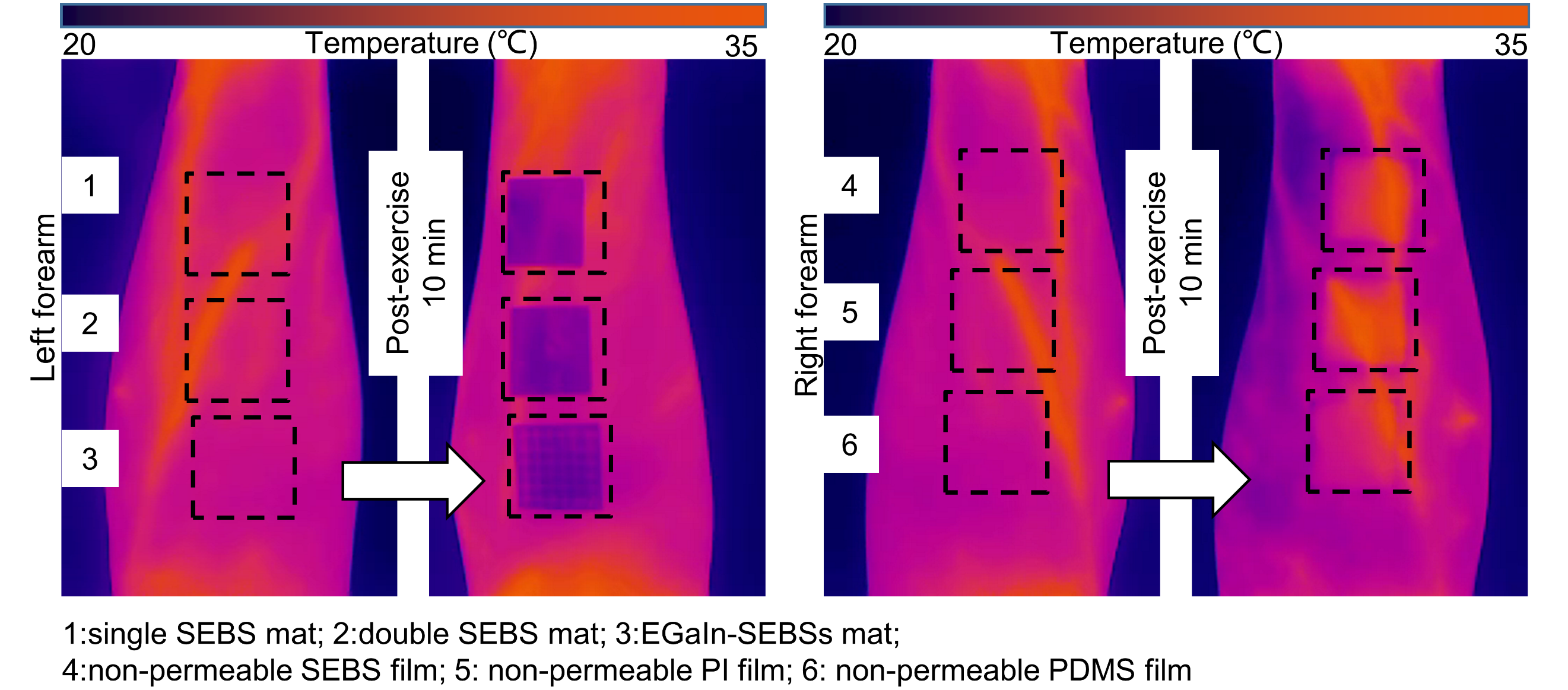


**Fig. S11** Infrared images showing the thermal monitoring using the different materials on the forearms of the volunteer


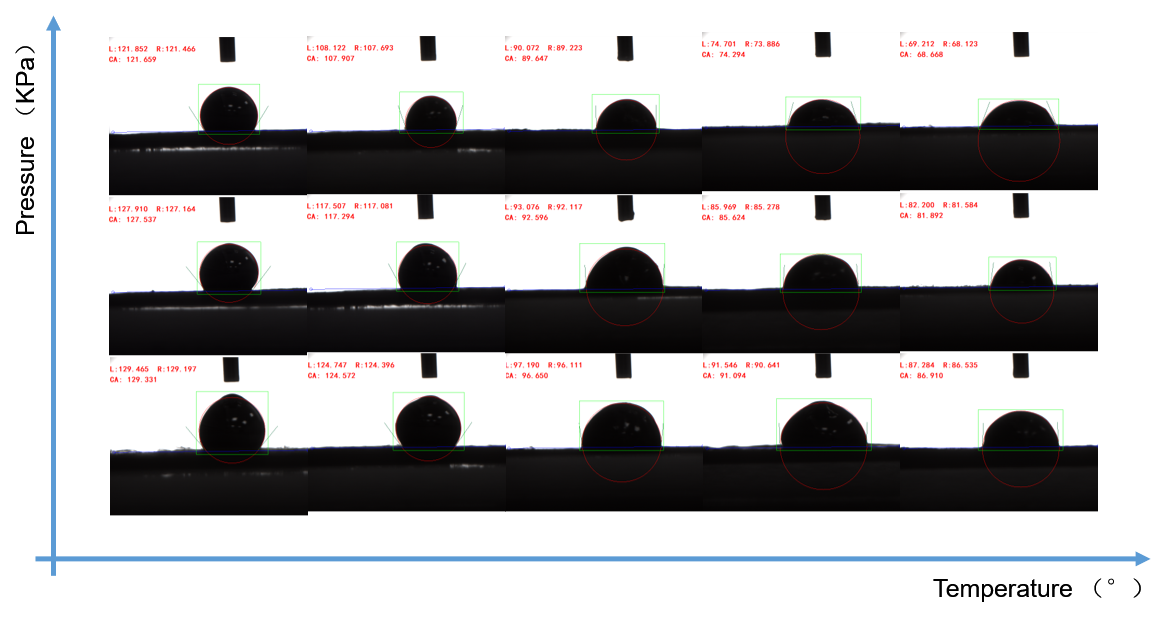


**Fig. S12** The contact angle of liquid metal on the surface of SEBS fibre mat before and after hot stamping treatment with different parameters


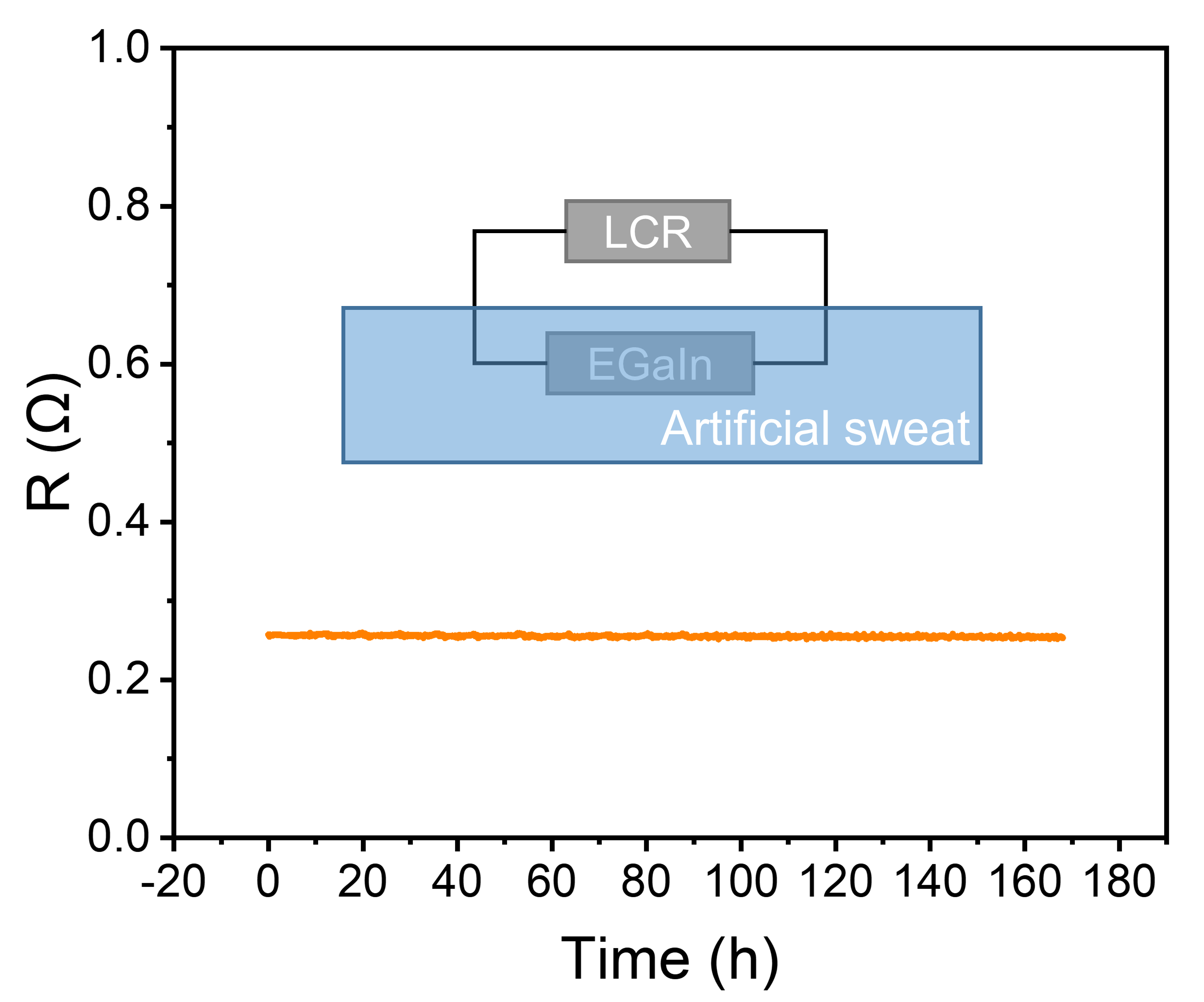


**Fig. S13** Resistance variation of flexible electronic devices in artificial sweat (NaCl, 0.9%) over a one-week period


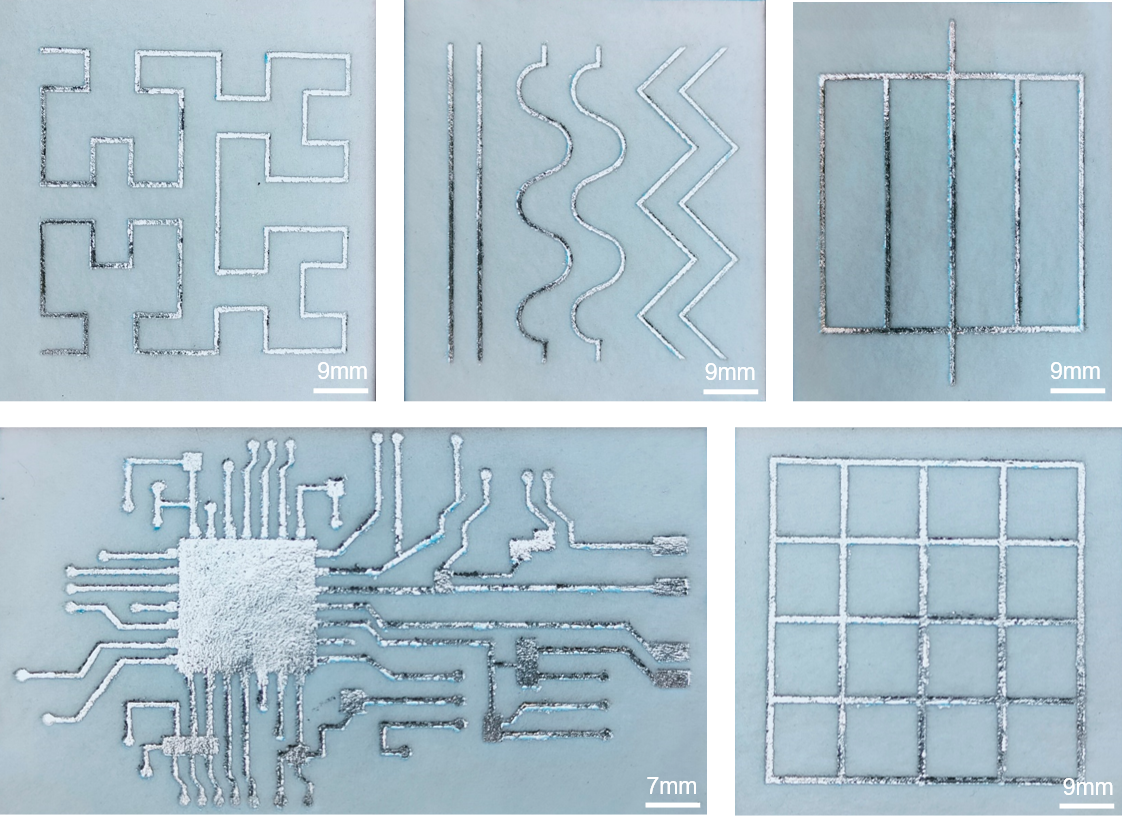


**Fig. S14** Optical image of liquid metal pattern on SEBS fiber mat


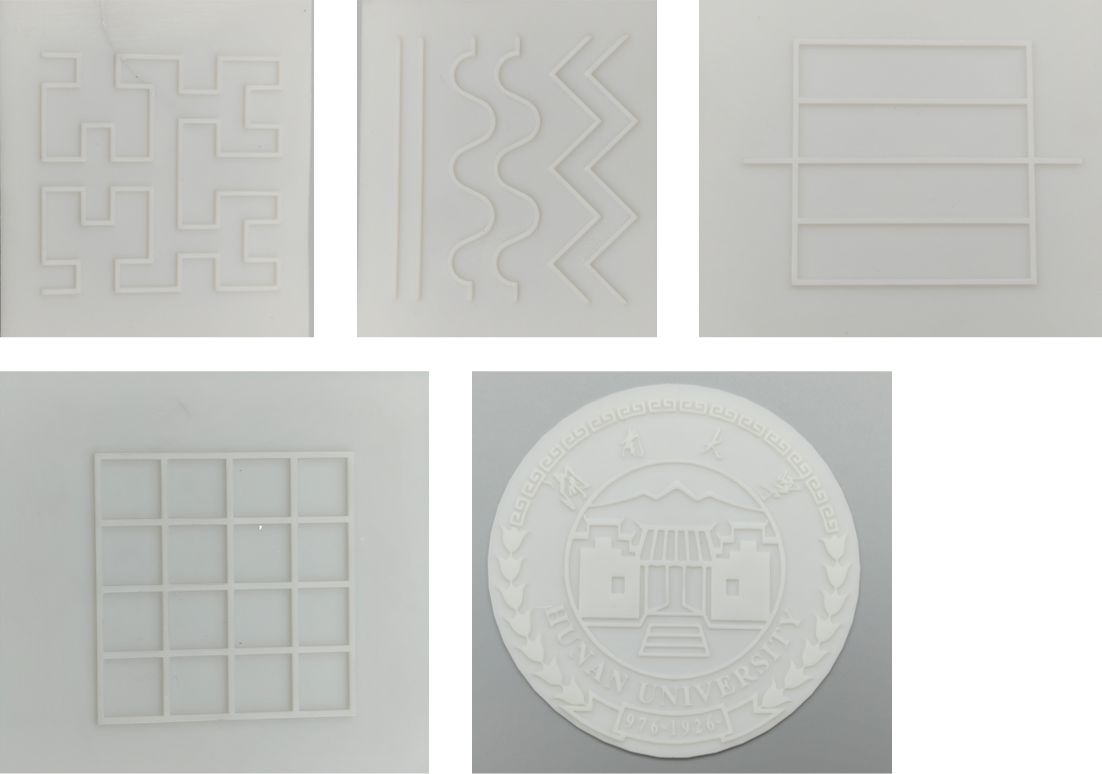


**Fig. S15** The imprinting mold used for patterning SEBS films


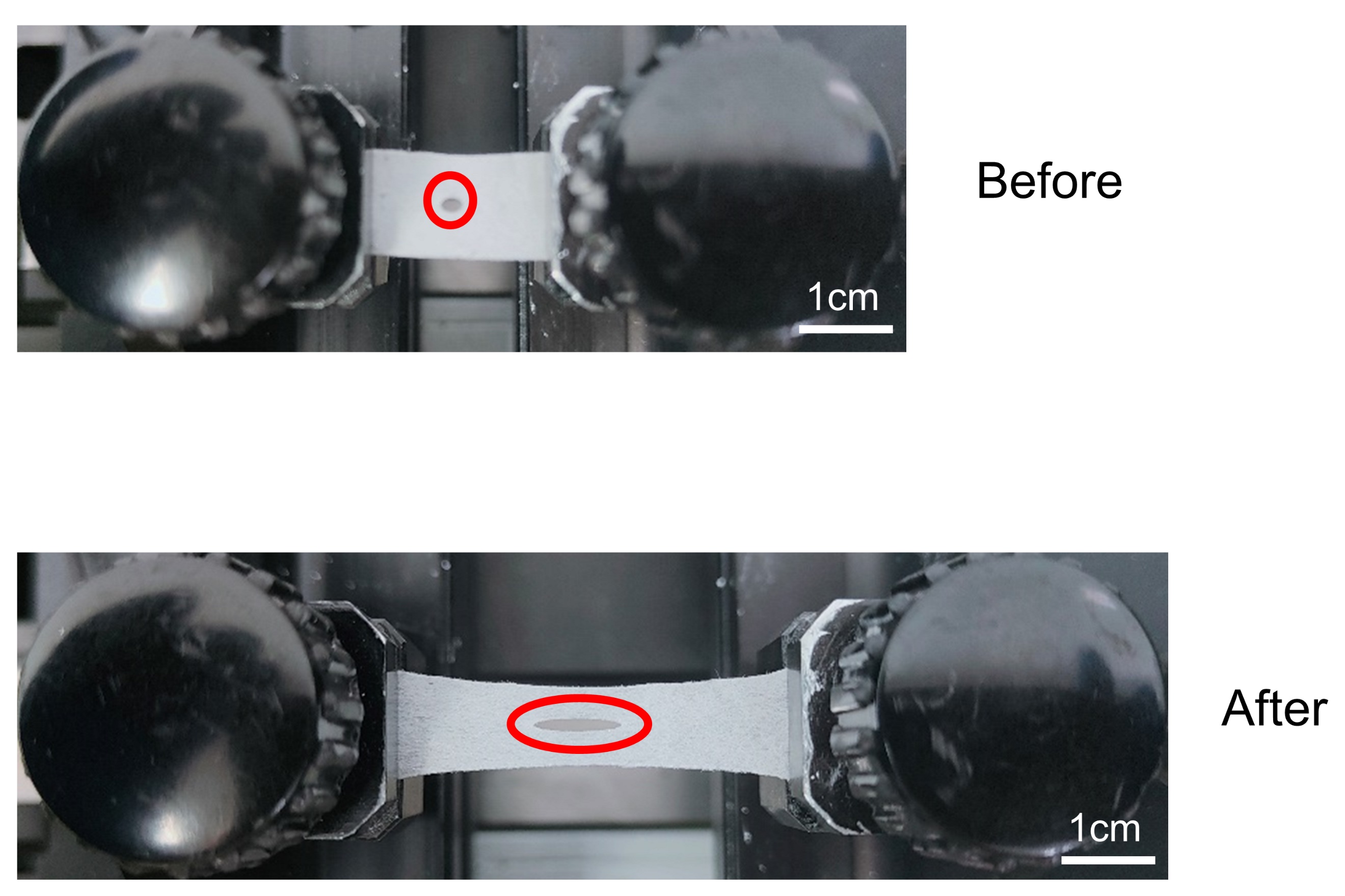


**Fig. S16** Deformation diagram of interlayer interconnected through pores of SEBS fibre mat before and after stretching at 100% tensile strain


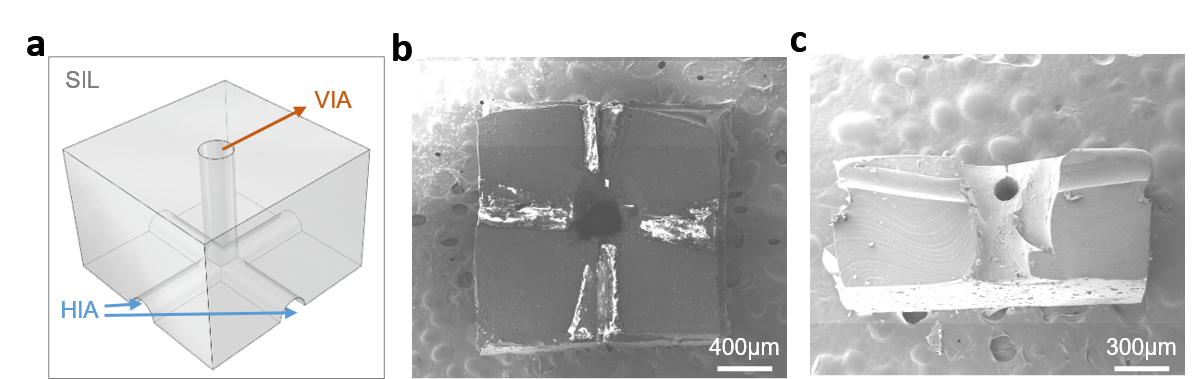


**Fig. S17** Strain-isolating layer (SIL). **a** 3D model diagram of SIL. **b** SEM images of SIL. **c** Cross-sectional SEM images of SIL


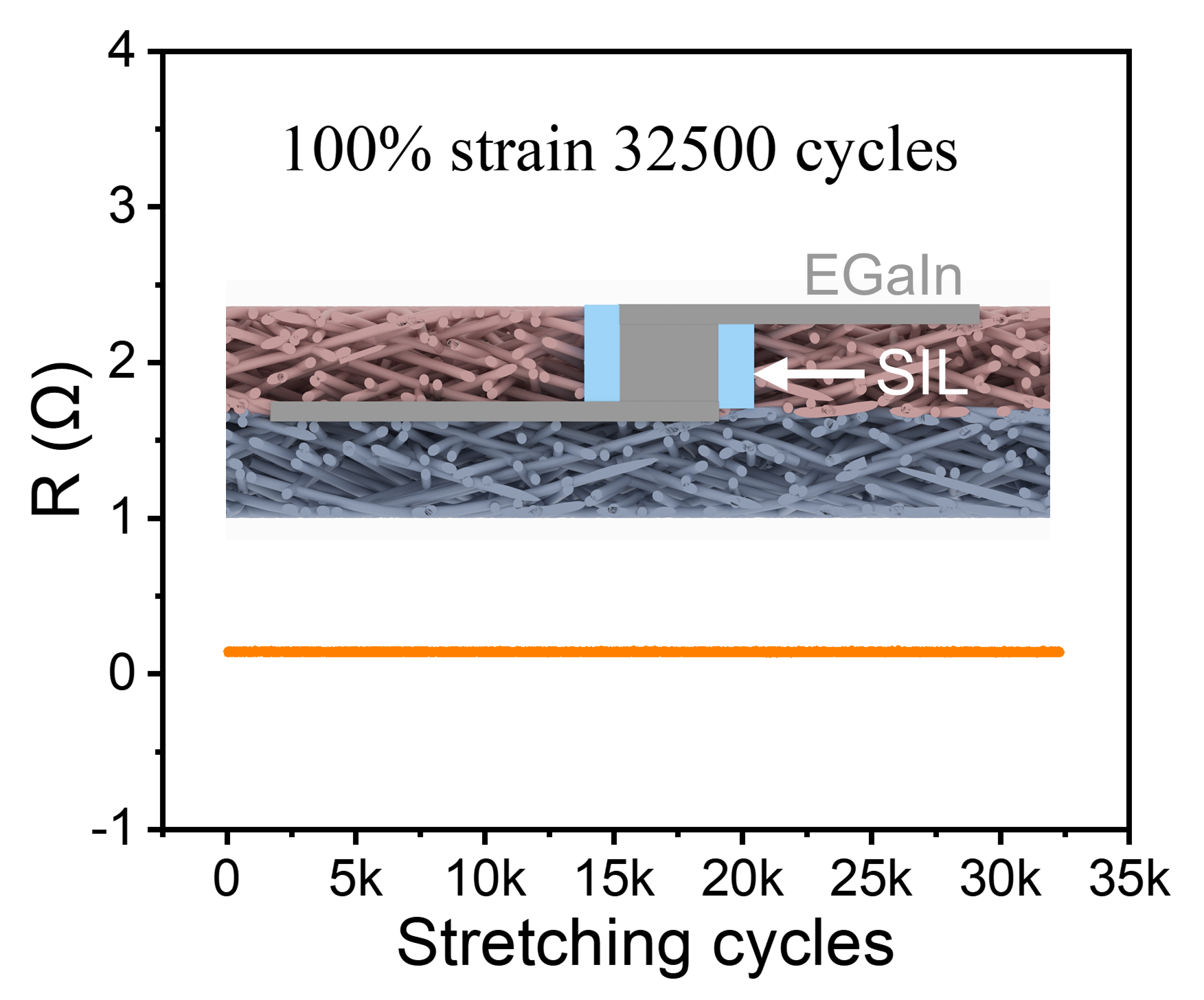


**Fig. S18** The strain isolator incorporating a 3D LM circuit demonstrates highly stable resistance under 100% strain over 32500 stretching cycles


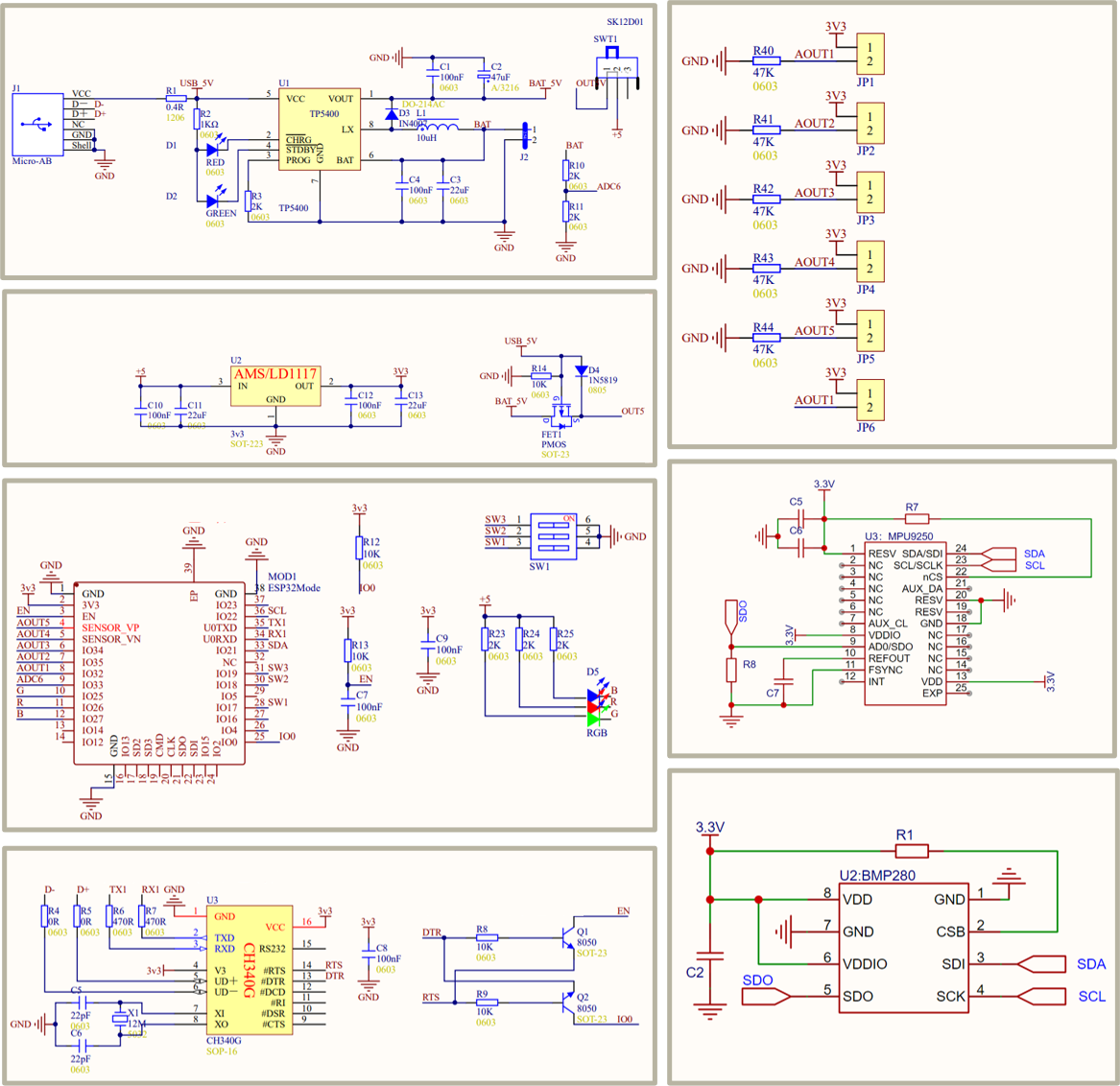


**Fig. S19** Summary of the circuit design for the 3D permeable electronic platform


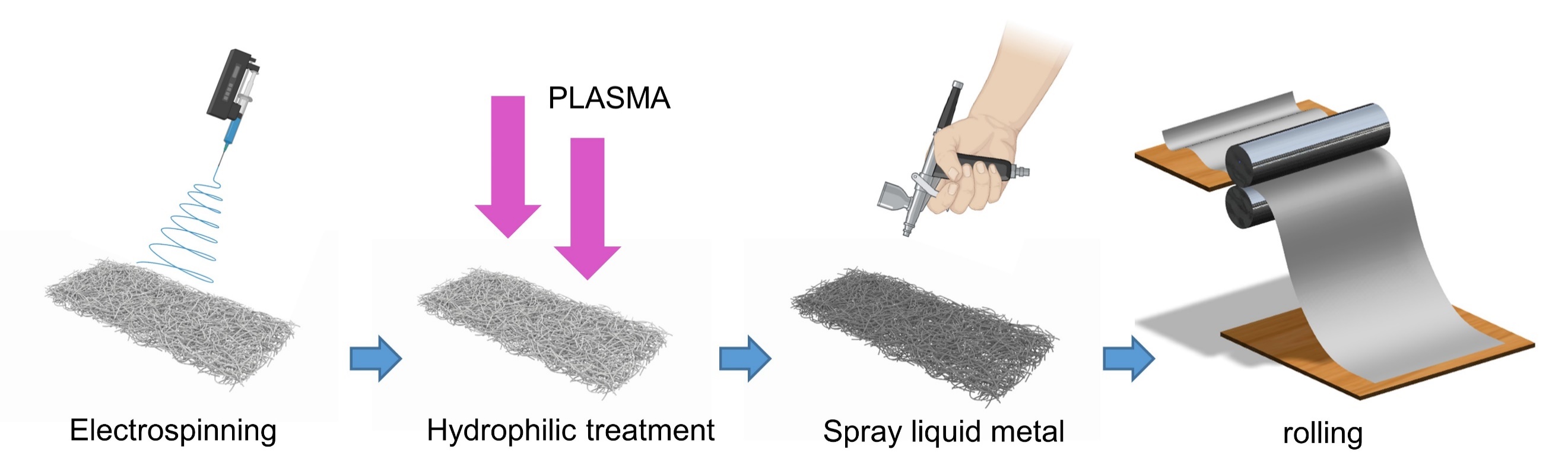


**Fig. S20** The production process of stretchable and breathable strain sensors


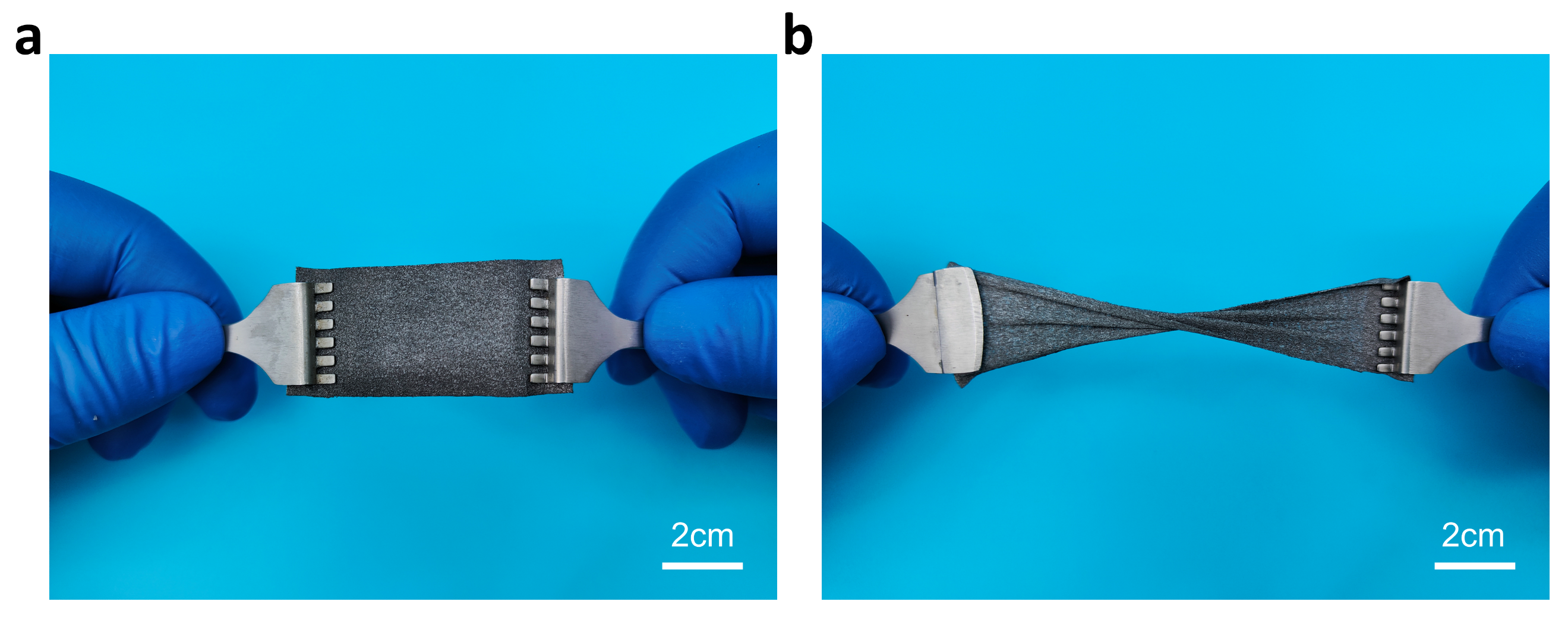


**Fig. S21** Optical diagram of strain sensor. **a** Optical diagram of strain sensor without stretching. **b** Optical diagram of strain sensor stretching and twisting


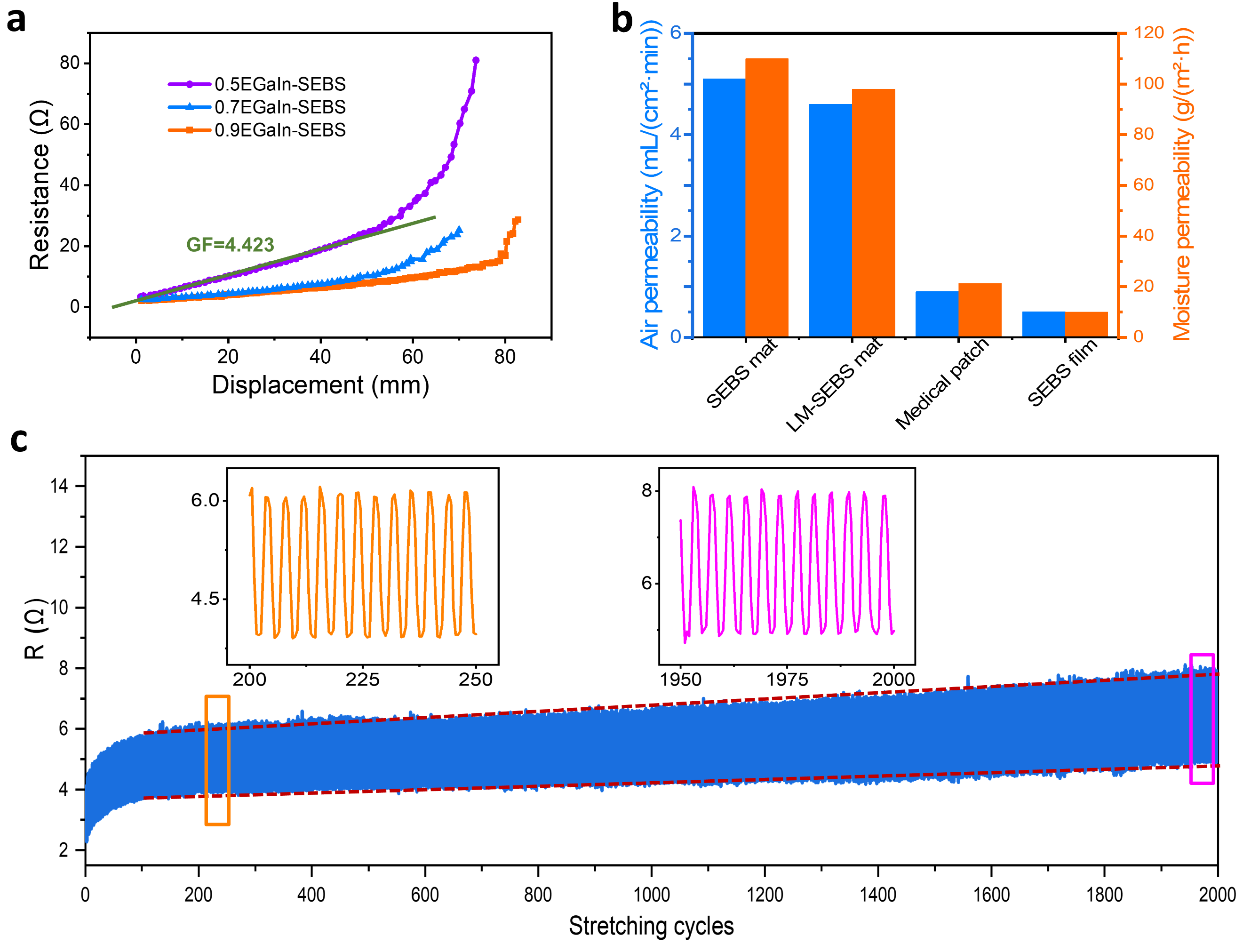


**Fig. S22** Characterization of flexible and stretchable breathable strain sensors. **a** The variation of resistance of strain sensors with different contents of liquid metals under different strain conditions. **b** Comparison of air permeability and moisture permeability between strain sensors and other common thin films. **c** Durability measurements under the tensile strain of 100%


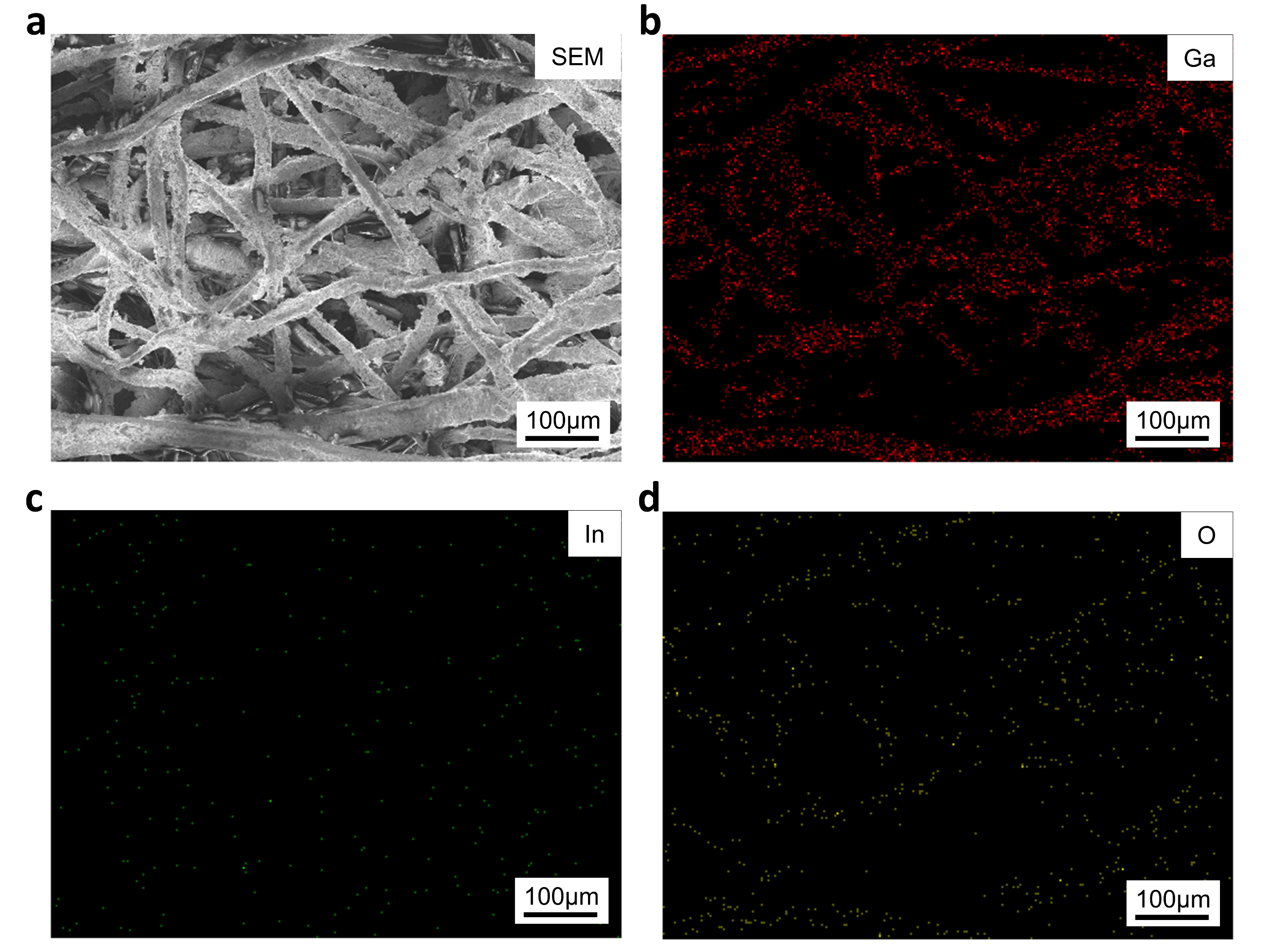


**Fig. S23 a** Surface scanning electron microscopy (SEM) images of strain sensor after multiple stretching cycles. **b c d** Energy dispersive X-ray energy spectrum (EDS) of strain sensor after multiple stretching cycles


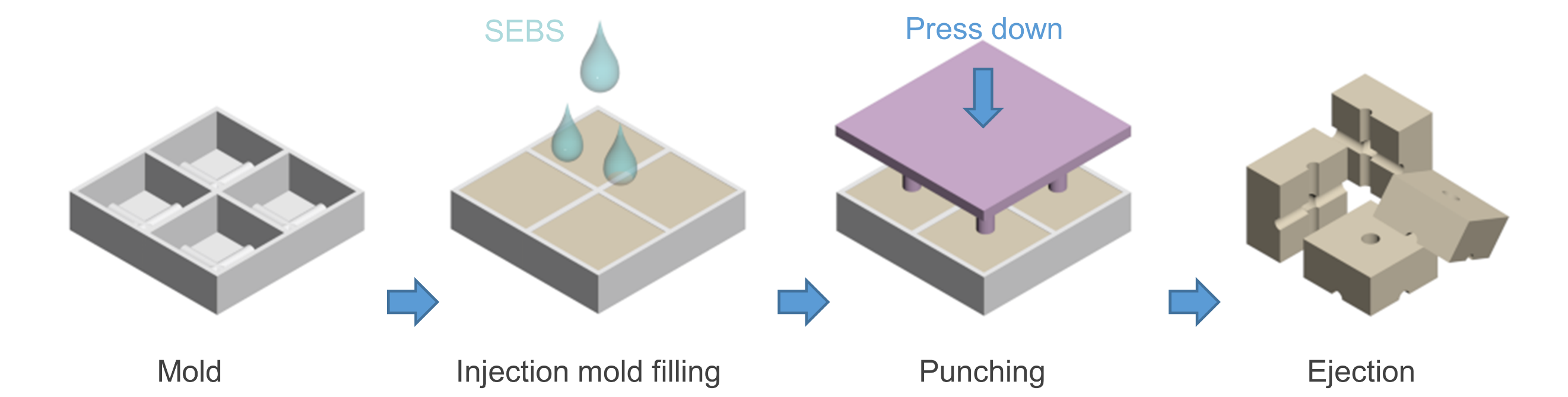


**Fig. S24** Schematic diagram of the production process of strain-isolating layer (SIL)


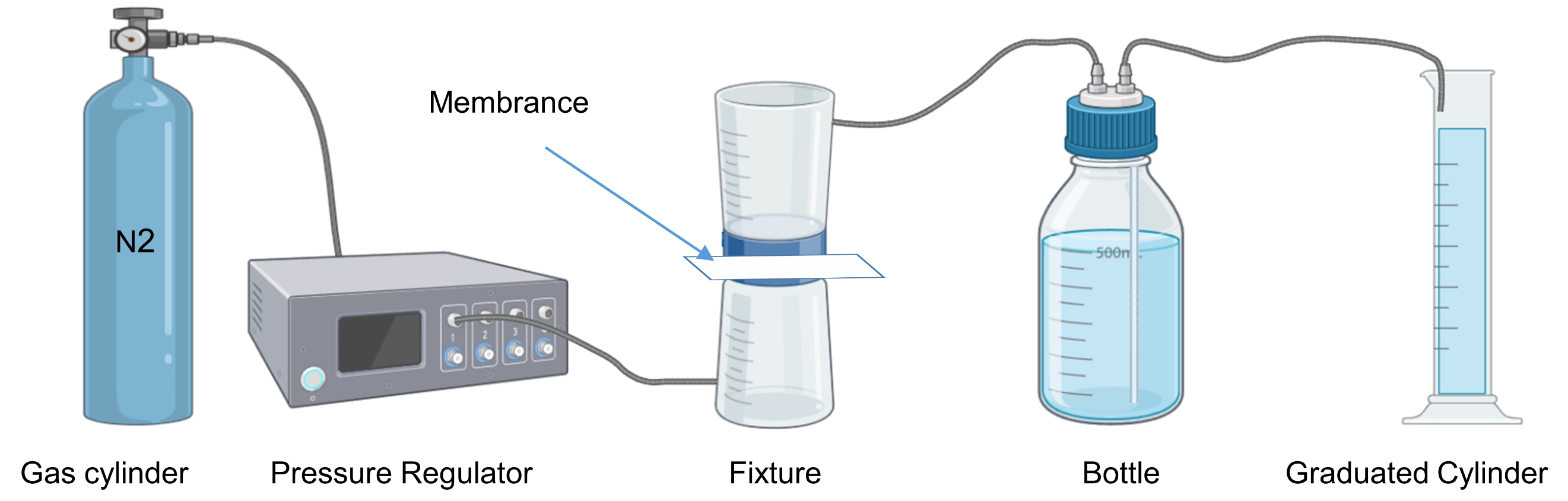


**Fig. S25** A homemade air permeability measurement system. The system consists of a gas cylinder, a pressure regulator, a detachable clamp, a gas collection bottle, and a graduated cylinder. The gas cylinder supplies nitrogen to the system as the gas source. The pressure regulator controls the pressure difference across the tested membrane and provides precise pressure feedback. The clamp secures the membrane under test. The graduated cylinder measures the volume of gas passing through the membrane.


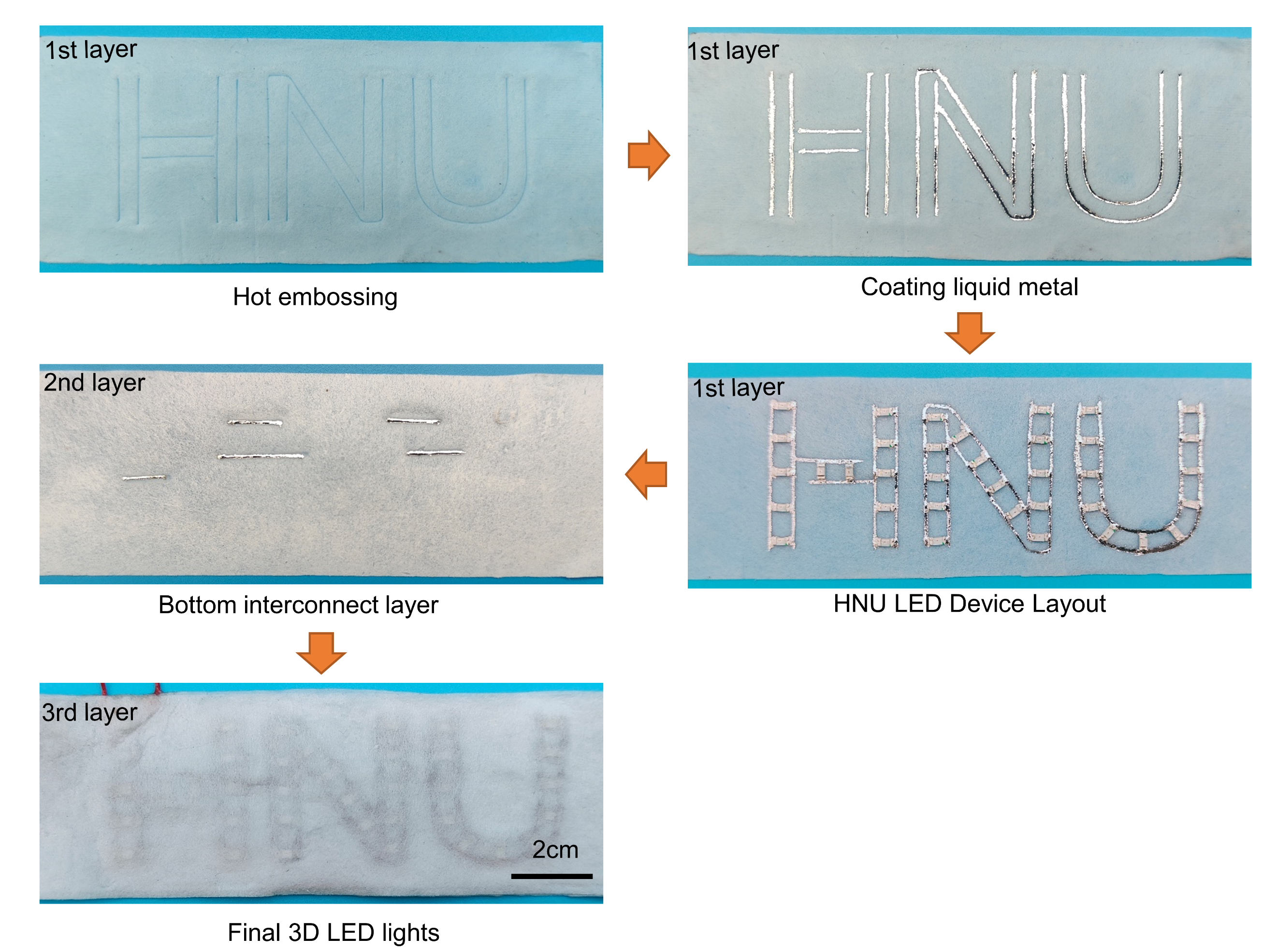


**Fig. S26** The production process of 3D stacked LED circuits

**Table S1** Summary of state-of-the-art integrated flexible and stretchable electronic systems

| **Strategy** | **Layout** | **Components** | **Integration Density (****/cm²)** | **Permeability (****g/m^2^/day))** | **Pattern Resolution (μm)** | **Mechanical Durability (Cycles)** | **Ref.** |
| --- | --- | --- | --- | --- | --- | --- | --- |
| **Thermal imprinting-SEBS/LM** | **Multilayer** | **140** | **7.9** | **2520** | **50** | **(100% strain)**  **32500** | **This work** |
| Photolithography-SBS/LM | Multilayer | 64 | 5.33 | 676 | 50 | (1500% strain)  1000 | [S1] |
| laser cutting- PI/Au | Single layer | N.A. | N.A. | N.A. | 50 | N.A. | [S2] |
| Chip-less wireless eskin | Single layer | N.A. | 1.99 | N.A. | N.A. | N.A. | [S3] |
| Photolithography-PP/Cu | Single layer | 45 | 0.45 | 561 | 100 | N.A. | [S4] |
| E-textile | Single layer | N.A. | N.A. | N.A. | N.A. | N.A. | [S5-S6] |
| Textile PCB | Multilayer | N.A. | N.A. | N.A. | N.A. | N.A. | [S7] |
| Photolithography-PDMS/Au | Single layer | N.A. | 4 | N.A. | 50 | (30% strain)  20000 | [S8] |
| Solidified LM | Multilayer | 22 | 3.273 | Impermeable | N.A. | (bending)  20000 | [S9] |
| Printing-PU/LM | Multilayer | 38 | 1.9 | Impermeable | N.A. | (100% strain)  15000 | [S10] |
| Photolithography-PI/ Cu | Multilayer | 42 | 37.834 | Impermeable | N.A. | (50% strain)  2000 | [S11] |
| Laser patterning-SIS/LM | Single layer | 60 | N.A. | Impermeable | 200 | (100% strain)  1000 | [S12] |
| Laser cutting mask | Multilayer | 7 | 0.1609 | Impermeable | N.A. | (100% strain)  1500 | [S13] |
| Serpentine circuits/silicone | Single layer | 31 | 2.849 | Impermeable | 50 | N.A. | [S14] |
| 3D integrated electronics | Multilayer | 56 | 9.824 | Impermeable | N.A. | (10% strain)  2000 | [S15] |
| Photolithography-PI/ Cu | Single layer | 50 | N.A. | Impermeable | 50 | N.A. | [S16] |

# N/A, not available.

**Table S2** The detailed parameters of the CNN model

| No | Layer Type | Filters/Units | Kerne/Pool Size | Stride | Input Size | Output Size |
| --- | --- | --- | --- | --- | --- | --- |
| 1 | Conv2D | 50 | (1,25) | (1,2) | (1,8,150) | (50,8,75) |
| 2 | Activation(ReLU) | - | - | - | (50,8,75) | (50,8,75) |
| 3 | MaxPool2D | - | (1,2) | (1,2) | (50,8,75) | (50,8,37) |
| 4 | Conv2D | 100 | (1,15) | (1,2) | (50,8,37) | (100,8,18) |
| 5 | Activation(ReLU) | - | - | - | (100,8,18) | (100,8,18) |
| 6 | MaxPool2D | - | (1,2) | (1,2) | (100,8,18) | (100,8,9) |
| 7 | Flatten | - | - | - | (100,8,9) | 7200 |
| 8 | Dense | - | - | - | 7200 | 512 |
| 9 | Activation(ReLU) | - | - | - | 512 | 512 |
| 10 | Dropout | - | - | - | 512 | 512 |
| 11 | Dense | - | - | - | 512 | 26 |
| 12 | Softmax | - | - | - | 26 | 26 |

**Table S3** Comparison with other wearable haptic interfaces on hands

| Strategy | Number of sensor units | Air Permeability (mm/s^-1^) | Moisture Permeability (g/m^2^/day) | Stretchability (%) | Recognition accuracy (%) | Refs. |
| --- | --- | --- | --- | --- | --- | --- |
| TENG | 4 | N/A | N/A | N/A | 92% | [S17] |
| Strain sensors | 5 | N/A | N/A | 100% | 98.8% | [S18] |
| Pressure Sensors | 5 | N/A | N/A | N/A | 96.8% | [S19] |
| TENG | 5 | N/A | N/A | 578.9% | 95.7% | [S20] |
| Tactile sensing | 128 | 40.3~46.3 mm/s^-1^ | 657.9~ 692.1 | 500 | 94.2% | [S21] |
| TENG | 5 | N/A | N/A | N/A | 98.5% | [S22] |
| Fiber | 5 | N/A | N/A | N/A | 98.63% | [S23] |
| TENG | 8 | N/A | N/A | N/A | 92.6% | [S24] |
| Strain sensors | 5 | N/A | N/A | 96.8% | 96.8% | [S25] |
| Capacitive sensor | 6 | N/A | N/A | N/A | 96.63% | [S26] |
| **Strain sensors** | **8** | **5.09295 mL/(cm²·min)** | **2520** | **750%** | **98%** | **This work** |

# N/A, not available.

**Supplementary References**

- 1. Q. Zhuang, K. Yao, C. Zhang, X. Song, J. Zhou et al., Permeable, three-dimensional integrated electronic skins with stretchable hybrid liquid metal solders. Nature Electronics. **7**(7), 598-609 (2024). <https://doi.org/10.1038/s41928-024-01189-x>
  2. B. Zhang, J. Li, J. Zhou, L. Chow, G. Zhao et al., A three-dimensional liquid diode for soft, integrated permeable electronics. Nature **628**(8006), 84-92 (2024). <https://doi.org/10.1038/s41586-024-07161-1>
  3. Y. Kim, J. M. Suh, J. Shin, Y. Liu, H. Yeon et al., Chip-less wireless electronic skins by remote epitaxial freestanding compound semiconductors. Science **377**(6608), 859-864 (2022). <https://doi.org/10.1126/science.abn7325>
  4. P. Wang, X. Ma, Z. Lin, F. Chen, Z. Chen et al., Well-defined in-textile photolithography towards permeable textile electronics. Nat. Commun. **15**(1), 887 (2024). <https://doi.org/10.1038/s41467-024-45287-y>
  5. H. W. Choi, D.-W. Shin, J. Yang, S. Lee, C. Figueiredo et al., Smart textile lighting/display system with multifunctional fibre devices for large scale smart home and iot applications. Nat. Commun. **13**(1), 814 (2022). <https://doi.org/10.1038/s41467-022-28459-6>
  6. R. Lin, H.-J. Kim, S. Achavananthadith, Z. Xiong, J. K. W. Lee et al., Digitally-embroidered liquid metal electronic textiles for wearable wireless systems. Nat. Commun. **13**(1), 2190 (2022). <https://doi.org/10.1038/s41467-022-29859-4>
  7. Y. Yang, X. Wei, N. Zhang, J. Zheng, X. Chen et al., A non-printed integrated-circuit textile for wireless theranostics. Nat. Commun. **12**(1), 4876 (2021). <https://doi.org/10.1038/s41467-021-25075-8>
  8. G. S. Jeong, D.-H. Baek, H. C. Jung, J. H. Song, J. H. Moon et al., Solderable and electroplatable flexible electronic circuit on a porous stretchable elastomer. Nat. Commun. **3**(1), 977 (2012). <https://doi.org/10.1038/ncomms1980>
  9. G. Li, M. Zhang, S. Liu, M. Yuan, J. Wu et al., Three-dimensional flexible electronics using solidified liquid metal with regulated plasticity. Nature Electronics. **6**(2), 154-163 (2023). <https://doi.org/10.1038/s41928-022-00914-8>
  10. W. Lee, H. Kim, I. Kang, H. Park, J. Jung et al., Universal assembly of liquid metal particles in polymers enables elastic printed circuit board. Science. **378**(6620), 637-641 (2022). <https://doi.org/10.1126/science.abo6631>
  11. H. Song, G. Luo, Z. Ji, R. Bo, Z. Xue et al., Highly-integrated, miniaturized, stretchable electronic systems based on stacked multilayer network materials. Sci Adv. **8**(11), eabm3785 (2022). <https://doi.org/10.1126/sciadv.abm3785>
  12. P. A. Lopes, B. C. Santos, A. T. de Almeida, M. Tavakoli. Reversible polymer-gel transition for ultra-stretchable chip-integrated circuits through self-soldering and self-coating and self-healing. Nat Commun. **12**(1), 4666 (2021). <https://doi.org/10.1038/s41467-021-25008-5>
  13. S. Liu, D. S. Shah, R. Kramer-Bottiglio. Highly stretchable multilayer electronic circuits using biphasic gallium-indium. Nature Materials. **20**(6), 851-858 (2021). <https://doi.org/10.1038/s41563-021-00921-8>
  14. H. U. Chung, B. H. Kim, J. Y. Lee, J. Lee, Z. Xie et al., Binodal, wireless epidermal electronic systems with in-sensor analytics for neonatal intensive care. Science. **363**(6430), eaau0780 (2019). <https://doi.org/10.1126/science.aau0780>
  15. Z. Huang, Y. Hao, Y. Li, H. Hu, C. Wang et al., Three-dimensional integrated stretchable electronics. Nature Electronics. **1**(8), 473-480 (2018). <https://doi.org/10.1038/s41928-018-0116-y>
  16. K.-I. Jang, K. Li, H. U. Chung, S. Xu, H. N. Jung et al., Self-assembled three dimensional network designs for soft electronics. Nat Commun. **8**(1), 15894 (2017). <https://doi.org/10.1038/ncomms15894>
  17. M. Guo, Y. Xia, J. Liu, Y. Zhang, M. Li et al., Wearable pressure sensor based on triboelectric nanogenerator for information encoding, gesture recognition, and wireless real-time robot control. Adv Funct Mater. **35**(22), 2419209 (2025). <https://doi.org/10.1002/adfm.202419209>
  18. C. Wu, S. Zhang, Y. Wang, F. Wang, T. Wang et al., 3d successive nanoscale interactions-driven mechanoreceptors with broad linear range and ultra-high sensitivity for efficient gesture recognition. Adv Funct Mater. **35**(34), 2500684 (2025). <https://doi.org/10.1002/adfm.202500684>
  19. Y. Zhou, S. Guo, Y. Zhou, L. Zhao, T. Wang et al., Ionic composite nanofiber membrane-based ultra-sensitive and anti-interference flexible pressure sensors for intelligent sign language recognition. Adv Funct Mater. **35**(29), 2425586 (2025). <https://doi.org/10.1002/adfm.202425586>
  20. L. Gao, J. Yang, Y. Zhao, X. Zhao, K. Zhou et al., Multilayer bionic tunable strain sensor with mutually non-interfering conductive networks for machine learning-assisted gesture recognition. Adv Funct Mater. **35**(11), 2416911 (2025). <https://doi.org/10.1002/adfm.202416911>
  21. K. Yao, Q. Zhuang, Q. Zhang, J. Zhou, C. K. Yiu et al., A fully integrated breathable haptic textile. Sci Adv. **10**(42), eadq9575 (2024). <https://doi.org/10.1126/sciadv.adq9575>
  22. L. Wang, X. Qi, C. Li, Y. Wang. Multifunctional tactile sensors for object recognition. Adv Funct Mater. **34**(49), 2409358 (2024). <https://doi.org/10.1002/adfm.202409358>
  23. Z. Zhou, K. Chen, X. Li, S. Zhang, Y. Wu et al., Sign-to-speech translation using machine-learning-assisted stretchable sensor arrays. Nature Electronics. **3**(9), 571-578 (2020). <https://doi.org/10.1038/s41928-020-0428-6>
  24. P. Tan, X. Han, Y. Zou, X. Qu, J. Xue et al., Self-powered gesture recognition wristband enabled by machine learning for full keyboard and multicommand input. Adv Mater. **34**(21), 2200793 (2022). <https://doi.org/10.1002/adma.202200793>
  25. T. Zhang, Y. Ding, C. Hu, M. Zhang, W. Zhu et al., Self-powered stretchable sensor arrays exhibiting magnetoelasticity for real-time human–machine interaction. Adv Mater. **35**(50), 2203786 (2023). <https://doi.org/10.1002/adma.202203786>
  26. A. Cheng, X. Li, D. Li, Z. Chen, T. Cui et al., An intelligent hybrid-fabric wristband system enabled by thermal encapsulation for ergonomic human-machine interaction. Nat Commun. **16**(1), 591 (2025). <https://doi.org/10.1038/s41467-024-55649-1>
